# Effects of repeated alcohol abstinence on within-subject prefrontal cortical gene expression in rhesus macaques

**DOI:** 10.3389/adar.2024.12528

**Published:** 2024-04-26

**Authors:** Robert Hitzemann, Lina Gao, Suzanne S. Fei, Karina Ray, Katinka A. Vigh-Conrad, Tamara J. Phillips, Robert Searles, Rita P. Cervera-Juanes, Rupak Khadka, Timothy L. Carlson, Steven W. Gonzales, Natali Newman, Kathleen A. Grant

**Affiliations:** ^1^ Department of Behavioral Neuroscience, Oregon Health and Science University, Portland, OR, United States; ^2^ Portland Alcohol Research Center, Oregon Health and Science University, Portland, OR, United States; ^3^ Veterans Affairs Portland Health Care System, Portland, OR, United States; ^4^ Bioinformatics and Biostatistics Core, Oregon National Primate Research Center, Oregon Health and Science University, Beaverton, OR, United States; ^5^ Division of Genetics, Oregon National Primate Research Center, Oregon Health and Science University, Beaverton, OR, United States; ^6^ Integrated Genomics Laboratory, Oregon Health and Science University, Portland, OR, United States; ^7^ Division of Neuroscience, Oregon National Primate Research Center, Oregon Health and Science University, Beaverton, OR, United States

**Keywords:** rhesus macaque, abstinence from alcohol, prefrontal cortex, RNA-seq, ethanol

## Abstract

Male rhesus monkeys (*n* = 24) had a biopsy of prefrontal cortical area 46 prior to chronic ethanol self-administration (*n* = 17) or caloric control (*n* = 7). Fourteen months of daily self-administration (water vs. 4% alcohol, 22 h access/day termed “open-access”) was followed by two cycles of prolonged abstinence (5 weeks) each followed by 3 months of open-access alcohol and a final abstinence followed by necropsy. At necropsy, a biopsy of Area 46, contralateral to the original biopsy, was obtained. Gene expression data (RNA-Seq) were collected comparing biopsy/necropsy samples. Monkeys were categorized by drinking status during the final post-abstinent drinking phase as light (LD), binge (BD), heavy (HD) and very heavy (VHD drinkers). Comparing pre-ethanol to post-abstinent biopsies, four animals that converted from HD to VHD status had significant ontology enrichments in downregulated genes (necropsy minus biopsy *n* = 286) that included immune response (FDR < 9 × 10^−7^) and plasma membrane changes (FDR < 1 × 10^−7^). Genes in the immune response category included *IL16* and *18*, *CCR1*, *B2M*, *TLR3*, *6* and *7*, *SP2* and *CX3CR1*. Upregulated genes (*N* = 388) were particularly enriched in genes associated with the negative regulation of MAP kinase activity (FDR < 3 × 10^−5^), including *DUSP 1*, *4*, *5*, *6* and *18*, *SPRY 2*, *3*, and *4*, *SPRED2*, *BMP4* and *RGS2*. Overall, these data illustrate the power of the NHP model and the within-subject design of genomic changes due to alcohol and suggest new targets for treating severe escalated drinking following repeated alcohol abstinence attempts.

## Introduction

AUD is diagnosed as a pattern of excessive alcohol consumption with repeated cycles of abstinence followed by resumption of heavy drinking, which in turn leads to negative psychological, physical, and social consequences [1]. This phenotype has been captured in a rhesus monkey model of alcohol self-administration using repeated cycles of chronic voluntary ethanol drinking and prolonged abstinence [2]. In this protocol, subjects are first induced to drink water and then ethanol (4% w/v) under a schedule-induced polydipsia (SIP) procedure. After induction, the monkeys given “open access” conditions for 22 h/day to choose between 45 ethanol. Given the voluntary nature of self-administration, the monkeys show a wide spectrum of individual differences in their daily intake ranging from an average of 0.3 g/kg/day up to 4 g/kg/day [3]. Based on mathematical modeling procedures and using data from six cohorts of monkeys with daily 22 h access to ethanol, four categorical levels of drinking were identified: light drinker (LD), binge drinker (BD), heavy drinker (HD) and very heavy drinker (VHD) [3, 4]. When the drinking protocol was extended to include three cycles of approximately 4–5 weeks of alcohol abstinence followed by 12–14 weeks of post-abstinence open-access conditions, alcohol intake generally increased following the reintroduction of alcohol (i.e., modeling resumption of heavy drinking) and gradually returned to pre-abstinence levels in all but a few monkeys [2]. The biological and behavioral features of this repeated abstinence protocol are described elsewhere [2, 5–7]. Overall, this repeated abstinence protocol has translational value for understanding the effects of cyclical chronic drinking and abstinence on subsequent drinking patterns.

The dorsolateral prefrontal cortex (DLPFC) is implicated in the return to heavy drinking in subjects with AUD, particularly in terms of volumetric analyses with magnetic resonance imaging (MRI) predicting sustained abstinence [8]. Indeed, subjects who returned to heavy drinking after 4 weeks of abstinence showed recovery of frontal cortical volume compared to subjects who maintained abstinence [8]. This suggests that atrophy of the DLPFC seen in relapsing AUD is due to underlying aberrant neuroadaptation to chronic heavy drinking. To address the molecular adaptations of the DLPFC in abstinence and just prior to resumption of heavy drinking, the present study utilized a biopsy of PFC area 46 before and following the protocol described above that ended with a final abstinence period (i.e., at necropsy). Rodent, nonhuman primate (NHP) and human postmortem studies have all clearly established the alignment of the brain transcriptome with ethanol consumption [9–13]. Rodent studies have been helpful in untangling risk genes and gene networks from those associated with the consequences of excessive ethanol exposure, as well as short-term abstinence (72 h) [14–18]. However, relatively little attention has been focused on the effects of protracted and/or repeated abstinence following chronic consumption. In addition, the rodent prefrontal cortex is underdeveloped compared to the primate brain. Thus, the current study offers a unique dataset addressing primate brain cortical adaptations and remodeling in abstinence from chronic alcohol consumption and just prior to resumption of heavy drinking. Finally, the data presented here offer a novel opportunity to examine how chronic ethanol exposure and repeated abstinence affect the NHP cortical transcriptome over a critical period of synaptic maturation, i.e., from late adolescence to young adulthood.

## Methods and materials

### Animals

Two replicate cohorts, named cohorts 10 and 14 in the Monkey Alcohol and Tissue Research Resource [MATRR] database [19], each containing 12 young adult male rhesus macaques were housed in quadrant cages (0.8 m × 0.8 m × 0.9 m) with constant temperature (20°C–22°C), humidity (65%), and an 11/13-h light/dark cycle. At the time of assignment, animals were between the ages of 4 and 6 years. Animals had visual, auditory, and olfactory contact with other animals in the protocol and were physically paired with another monkey for 1–2 h/day. All animals were maintained on positive caloric (i.e., weight gain) and fluid balance throughout the experiment, and body weights were recorded weekly. All procedures were conducted in accordance with the Guide for the Care and Use of Laboratory Animals and the NIH PHS Policy on Humane Care and Use of Laboratory Animals and approved by the Oregon National Primate Research Center (ONPRC) Institutional Animal Care and Use Committee (Protocol #0756). Animals in both cohorts were chosen from the ONPRC breeding colony to avoid common parents or grandparents. Cohort 10 contained 4 controls and 8 ethanol drinkers, and cohort 14 contained 3 controls and 9 ethanol drinkers. Detailed drinking and physiological data are available for these cohorts online.^1^

### Ethanol subjects

Monkeys were trained to use operant drinking panels and underwent induction of ethanol self-administration in daily 16-h sessions using the SIP protocol as previously described [20]. Following induction, “open-access” conditions (22 h/day of a concurrent choice of drinking ethanol (4% w/v diluted in water or water) were scheduled every day for 60 weeks.). The details of the open-access protocol are discussed in Grant et al. [20], and the data modeling for drinking categories are presented in Baker et al. [3]. Ethanol and water intakes were recorded daily with a 0.5 s resolution, 22 h/day.

### Control subjects

Yoked control subjects were housed in the same room as the ethanol-drinking subjects and participated in all experimental manipulations. For the controls, the SIP and self-administration conditions were identical, with the exception that both spouts dispensed water. A maltose dextrin solution (10% in water) was given to the controls in a volume calorically matched to intake by a weight-matched ethanol monkey. The dextrin solution was given at the beginning of each daily session by attaching a bottle to the front of the housing cage.

### Abstinence

After daily open-access to ethanol, the first abstinence phase began. All independent variables remained constant except that the ethanol bottle was replaced with a second water bottle. Yoked controls no longer received daily maltodextrin during the abstinence phase. Abstinence 1 lasted for a total of 34 days, which included additional time for structural MRI scans (2 monkeys/day were scanned over the last week). At the end of the first abstinence, ethanol was again made available for the ethanol monkeys, and yoked maltose dextrin was reinstated for the control monkeys for approximately 11 weeks. Abstinence 2 followed and was 4 weeks for cohort 10 but extended to 7 weeks for cohort 14 in order to schedule and acquire fMRI (Table 1). After reinstating ethanol consumption for 14 weeks, abstinence 3 followed and was 34 days.

**TABLE 1:** Number of sessions in each experimental phase by cohort^a^.

Experimental phase	Cohort 10	Cohort 14
“12 months” open access	366	343
Post 12 months procedures	54	85
Total open-access prior to abstinence	420	428
Abstinence 1	34	34
Post abstinence 1 open access	83	76
Abstinence 2	27	41^b^
Post abstinence 2 open access	102	102
Abstinence 3	34	38

^a^ The table indicates the average time each cohort spent in each of the experimental phases. The differences between cohorts 10 and 14 were largely due to scheduling issues.

^b^ Phase extended to collect MRI data.

### Biopsy/necropsy samples

MRI was used to obtain the stereotaxic coordinates for prefrontal cortical area 46 biopsies from the contralateral dominant hemisphere (determined by handedness). Tissue samples (35–50 mg wet weight) were taken under anesthesia (ketamine HCl 15 mg/kg; intramuscular) prior to ethanol exposure (hereafter noted as the Biopsy samples) and at necropsy; the necropsy samples were taken contralateral to the baseline Biopsy samples and were taken immediately before perfusion. Necropsy occurred at the end of the third abstinence period. Samples were immediately flash frozen in liquid nitrogen in a 2 mL cryovial and stored at −80°C for future processing. The pre-alcohol and necropsy biopsy samples were processed simultaneously using a standard Qiagen AllPrep DNA/RNA/miRNA Universal Kit protocol.

### RNA-seq

Libraries were prepared using either the TruSeq RNA Sample Preparation kit (Illumina) or the TruSeq Stranded mRNA sample preparation kit (Illumina). Sequencing was performed on either a HiSeq 2500 (Illumina) or a NovaSeq 6000 (Illumina) at the Oregon Health and Science University Massively Parallel Sequencing Shared Resource. The quality of the raw sequencing files was evaluated using FastQC^2^ combined with MultiQC^3^ [21]. Trimmomatic [22] was used to remove any remaining Illumina adapters. Reads were aligned to Ensembl’s mmul10 along with its corresponding annotation, release 100. The program STAR [23] (v2.7.3a) was used to align the reads to the genome utilizing these parameters: --outFilterMismatchNmax 3 --outFilterMultimapNmax 1. STAR has been shown to perform well compared to other RNA-seq aligners [24]. Since STAR utilizes the gene annotation file, it also calculates the number of reads aligned to each gene. RNA-SeQC [25] and another round of MultiQC were utilized to ensure that alignments were of sufficient quality.

Gene-level differential expression (DE) analysis was performed in open-source software R (R Core Team^4^). Gene-level raw counts were filtered to remove genes with low counts in many samples following published guidelines [26], normalized using the trimmed mean of M-values method (TMM) [27], and transformed to log-counts per million with associated observational precision weights using the voom [28] method. Gene-wise linear models suited to the experimental design were employed for differential expression analyses using limma with empirical Bayes moderation [29] and false discovery rate (FDR) adjustment [30]. More specifically, in Necropsy vs. Biopsy Comparisons, separate models comparing Necropsy vs. Biopsy all adjust for Cohort and account for correlation within animals. In biopsy-only comparisons, because of the expected high biological variation (unlike the necropsy vs. biopsy comparisons), these comparisons do not have each animal as its own control. Surrogate variable analysis (SVA) [31] was performed to identify and adjust for any unknown sources of variation. Separate models comparing specified groups all adjust for cohort and surrogate variables identified through SVA.

The details of how the data were analyzed and the discrete sample sizes have been integrated into the results (see below). DE genes are commonly defined using statistical thresholds; because the degrees of gene expression profile change are expected to be different between the comparisons, different practical statistical thresholds were used to define DE genes to facilitate the identification of biological implications through pathway analyses (see below). Here, we note that the ontologies for various groups of differentially expressed genes were determined using the GOrilla algorithm [32] with human annotations; transcripts meeting the threshold of one transcript per million were used as the background reference. The significance level was set at FDR (q) < 0.05. Pathway analyses were performed in IPA [33] (QIAGEN Inc.^5^). Additional analysis details are found in the Supplementary Datasheet S1 as “Cohort 10 and 14 RNA-seq DE analysis, v9.”

## Results

### Sample characteristics

**FIGURE 1:**
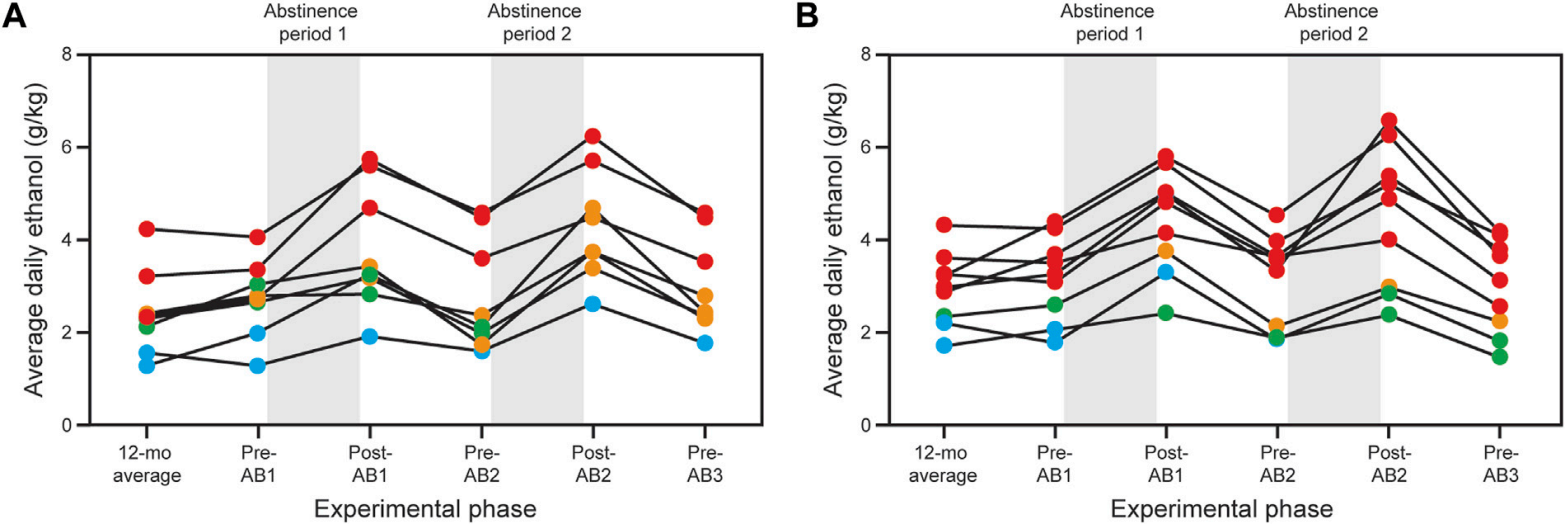
Effects of abstinence on ethanol consumption in cohort 10 **(A)** and cohort 14 **(B)**. Both cohorts consumed alcohol in a choice paradigm for 12–14 months as described in the Methods and Grant et al. [20]. The final month of choice consumption was used to designate the subjects as light (blue), binge (green), heavy (orange) and very heavy (red) drinkers [see Baker et al. (2012) for detailed definitions of the drinking categories]. Both cohorts underwent three 1-month abstinence periods; the first two were followed by 3 months of standard choice consumption. Animals were sacrificed at the end of the third abstinence period (not illustrated in the figure). Additional details are found in Table 1.

Twenty-four male rhesus macaques (7 controls and 17 drinkers) were entered into the analysis. Subjects were equally derived from 2 cohorts (10 and 14), separated experimentally by approximately 3 years. Biopsy/necropsy samples were obtained for all of the controls and 13 of the 17 drinkers. Four of the drinkers in cohort 10 underwent sham biopsy surgery; thus, only necropsy samples were obtained. The timeline for the progression of the subjects is illustrated in Figure 1. Additional details are found in Table 1. The data shown for the end of the initial 60 weeks of chronic ethanol access are average daily intakes over the last 30 days of open-access. The ethanol monkeys at this time point, were categorized as 3 LD, 5 BD, 4 HD and 5 VHD. MATRR subject IDs are found in the legend to Figure 1. There were no significant age differences among the groups. The drinking patterns after the second abstinence period revealed the following transitions from the end of the initial 60 weeks of open-access intakes: 4 BDs → HDs (# *10208, 10209*, 10211, 10251), 2 LDs → HDs (#*10213* 10246) and 4 HDs → VHDs (# 10212, 10244, 10247, 10249). Five VHDs remained VHDs (#10214, 10215, 10242, 10248, 10252). (Note: the subject IDs in italics are the subjects for whom only a necropsy sample was available.)

### Biopsy and necropsy independent comparisons

Three planned comparisons were made for the biopsy-only gene expression data: drinkers vs. controls, HD+VHD vs. LD+BD and VHD vs. HD. Drinking categories are those observed at the end of the period of voluntary consumption. The DE results for these comparisons are found in Supplementary Table S1. No significant DE genes were detected at the commonly used threshold of FDR < 0.2, suggesting minimal gene expression differences between these groups at biopsy. These data are also illustrated in Supplementary Data Sheet S1 as volcano plots and heatmap plots. Although this drinking protocol will not be repeated in future cohorts, biopsies from cohorts with different drinking protocols will be combined with these to increase sample sizes for a biopsy-only analysis in future studies. The drinkers vs. controls were also compared at necropsy; as indicated in Supplementary Table S1, there were no significant differences.

### Necropsy vs. biopsy comparisons

The following comparisons were made for the necropsy vs. biopsy data: controls, controls vs. drinkers, HDs → VHDs, VHDs and BDs → HDs. The necropsy drinking categories are those seen after the second abstinence period. The comparison for the subjects who remained VHDs was included since, as noted in Figure 1, abstinence also increased consumption in this group. The DE results for the necropsy vs. biopsy comparisons are also found in Supplementary Table S1. For the drinker comparisons and given the small sample sizes, the FDR was relaxed to 0.2. Volcano plots, heatmaps and venn diagrams are provided in Supplementary Data Sheet S1.

### Control comparison

**FIGURE 2:**
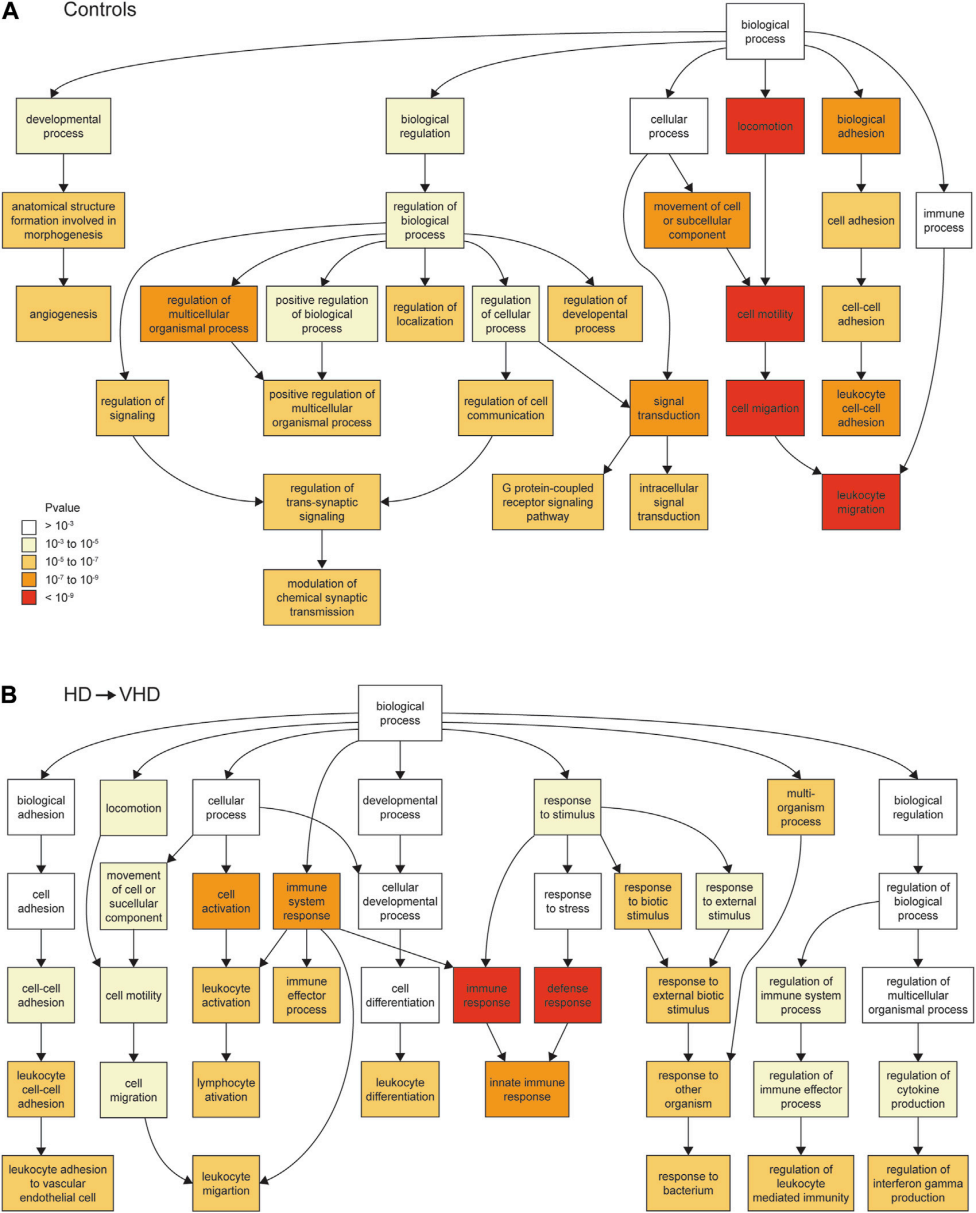
Biopsy/necropsy comparisons for controls [*N* = 7, **(A)**] and for HD → VHD [*N* = 4, **(B)**]. The ontologies in the figures are for the downregulated genes and in the ontology process category. The Gorilla algorithm (Aden et al. 2009) was used to detect the ontologies. The biopsy and necropsy samples differed temporally by approximately 3 years. The ontology categories are color coded according to the strength of the ontology **(A)**. Note the difference in the strength of the immune response ontology between the controls and the HD → VHD subjects.

The control (*N* = 7) data were collected over the period that spans late adolescence to early adulthood. Over this period, 269 genes were downregulated (necropsy < biopsy), and 456 were upregulated (necropsy > biopsy). The Gene Ontologies (GO) of these DE expressed genes are summarized in Supplementary Table S2. For the downregulated genes, the ontologies included the following: **process**: cell migration (FDR < 2 × 10^−8^); signal transduction (FDR < 5 × 10^−5^); cell adhesion (FDR < 1 × 10^−4^); **function**: transmembrane signaling receptor activity (FDR < 7 × 10^−5^); and **component**: plasma membrane part (FDR < 8 × 10^−11^). A schematic showing the relationships of the ontologies in the Process category is shown in Figure 2A. For the purpose of the latter discussion, we note here the detection of a weak (FDR < 0.01) immune response ontology (Figure 2A); genes in this ontology include *Icam1*, *Prkcz*, *Hyal1* and *3*, and *Tlr7.* Genes involved in signaling receptor activity included *P2ry13*, *P2ry12*, *Tlr7*, *Grm3* and *Htr6*. However, note that *P2ry12* and *13* are located in microglia and can be considered immune related genes. For the upregulated genes, the ontologies included the following: **process**: RNA processing (FDR < 4 × 10^−6^); **function**: nucleic acid binding (FDR < 2 × 10^−8^); and **component**: nuclear part (FDR < 1 × 10^−7^).

### HD → VHD comparison

For the animals (*N* = 4) that converted from HD to VHD status during the abstinence protocol, 286 genes were downregulated and 388 were upregulated. Ontologies (Supplementary Table S2) for the downregulated genes included the following. **Process**: immune response (FDR < 9 × 10^−7^); and **Component**: plasma membrane part (FDR < 1 × 10^−7^). Genes in the immune response category included *Il16* and *18*, *Ccr1*, *B2m*, *Tlr3*, *6* and *7*, *Sp2* and *Cx3cr1.* Genes in the plasma membrane category included the purinergic receptors noted above. Figure 2B provides a schematic of the process ontologies; a comparison of Figures 2A, B illustrates the marked downregulation of immune-related genes in the HD → VHD group. Enriched ontologies for the upregulated genes included the following. **Process:** regulation of RNA metabolic process (FDR < 3 × 10^−5^) and negative regulation of MAP kinase activity (FDR < 3 × 10^−5^); **Function**: transcription regulatory region DNA binding (FDR < 2 × 10^−3^) and **Component**: glutamatergic synapse (FDR < 4 × 10^−2^). The negative regulation of MAP kinase activity is a new ontology not seen in the control group. Genes in this group included *Dusp 1*, *4*, *5*, *6* and *18*, *Spry 2*, *3*, and *4*, *Spred2*, *Bmp4* and *Rgs2*. Genes in the glutamatergic synapse category largely encoded accessory synaptic proteins but did include *Grik3.*

### VHD → VHD comparison

As noted above, there were 5 VHDs that remained VHDs at the end of the abstinence protocols. One member of this group had only a necropsy sample that was included in the analyses. Fifty-eight genes were downregulated, and 94 were upregulated. For the downregulated genes, there were no significant (FDR < 0.05) GO terms. For the upregulated genes, significantly enriched ontologies included the following: **Process**: the negative regulation of MAP kinase activity (FDR < 2 × 10^−6^); and **Function**: protein kinase inhibitor activity (FDR < 1 × 10^−2^). Genes in the negative regulation of MAP kinase activity category were similar to those noted above and included *Dusp4*, *5* and *6*, *Rgs2*, *Spry2* and *3*, *Spred2* and *Bmp4*.

### BD → HD comparison

There were 4 BD subjects who converted to HD at the end of the abstinence protocol. Biopsy samples were available for only 2 of these subjects. Four hundred ten genes were downregulated, and 252 genes were upregulated (necropsy vs. biopsy). For the downregulated genes, significantly enriched ontologies included the following. **Process:** signal transduction (FDR < 8 × 10^−4^), defense response (FDR < 2 × 10^−2^); **Function:** signaling receptor activity (FDR < 2 × 10^−3^); and **Component**: plasma membrane part (FDR < 3 × 10^−11^). Genes in the signaling receptor activity category included *Arra2c*, *Ccr1*, *Gabrg1*, *Grin2c* and *Tlr7.* For the upregulated genes, there was only one significant ontology. **Process**: regulation of RNA splicing (FDR < 4 × 10^−2^).

### Controls vs. drinkers comparison

Controls and drinkers (*at biopsy and necropsy*) were compared. As illustrated in the venn diagrams and volcano plots (Supplementary Data Sheet S1), there were no significant differences between these groups.

### Ingenuity pathway analysis (IPA)

**FIGURE 3:**
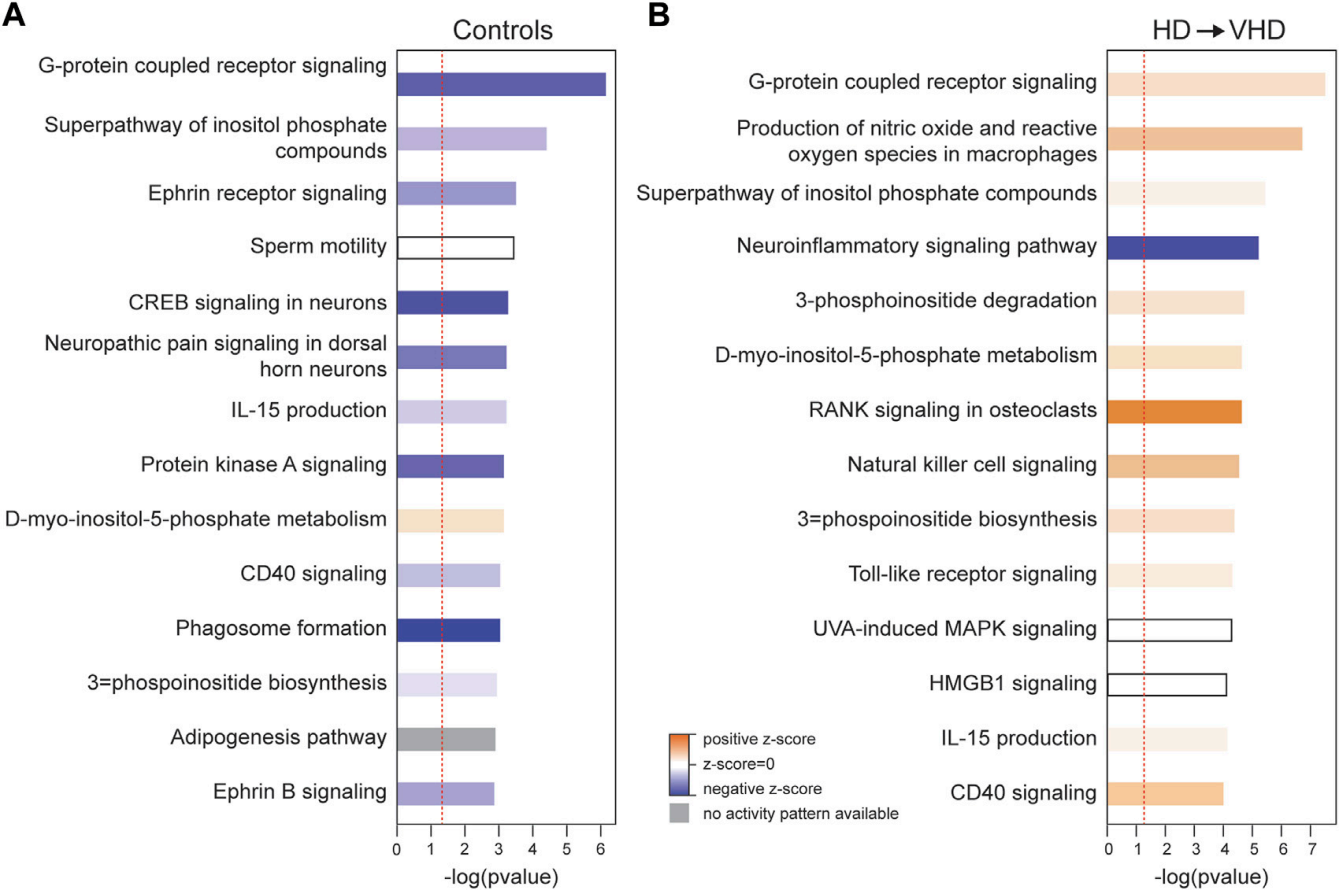
Ingenuity Pathway Analysis (IPA) of the biopsy/necropsy DE genes in the control **(A)** and HD→VHD **(B)** groups. Data entered into the analyses were both the up- and downregulated genes. The data presented are for the top 15 canonical pathways. Solid blue indicates pathways downregulated in the necropsy sample, and solid red indicates pathways upregulated in the necropsy sample. Similar to the data in Figure 2, IPA detected the downregulation of G-protein coupled receptor signaling in the controls and neuro-inflammatory signaling in the HD → VHD group.

**FIGURE 4:**
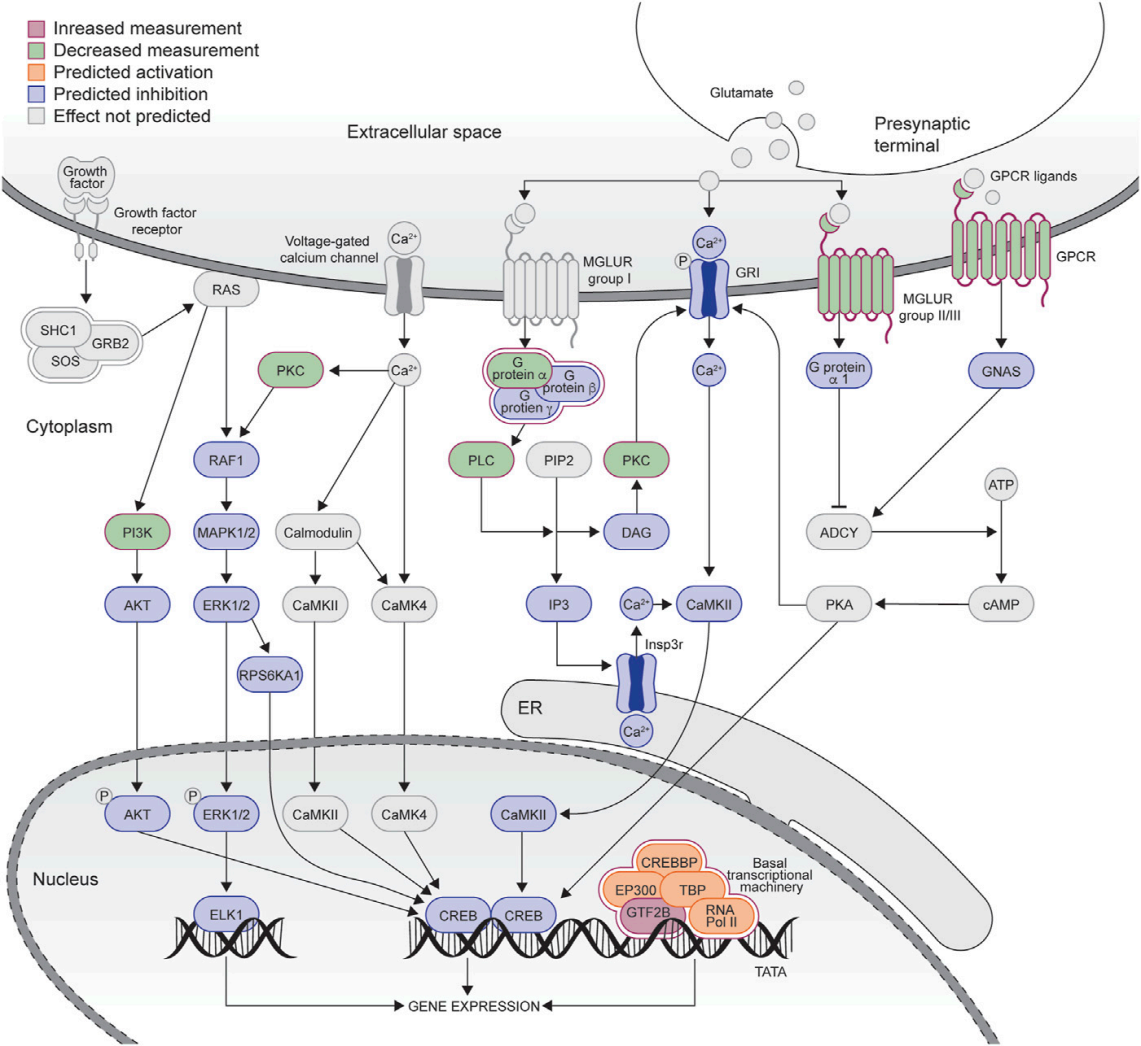
Graphical representation of the CREB signaling pathway downregulated in the control group (see Figure 3A). The graph was adapted from the graph generated by IPA. Blue coloring = downregulated genes, red coloring = upregulated genes. Green coloring = genes in the pathways that were not different between the biopsy/necropsy samples.

IPA was used to examine enriched pathways in the control and HD→VHD biopsies to necropsy DE genes. Figures 3A, B compare the top canonical pathways in each group (Z score <−2 or >2). For the controls, 4/14 pathways had a Z score <−2, i.e., these pathways were predicted to be downregulated in the necropsy sample. These pathways included G-Protein Coupled Receptor Signaling, CREB Signaling in Neurons, Protein Kinase A Signaling and Phagosome Formation. The CREB signaling pathway is illustrated in Figure 4. The DE genes in this pathway included five adhesion G-protein coupled receptors (B2, E5, G1, L4 and V1), 3 alpha G-protein subunits (11, 14 and Z), 5 G-protein coupled receptors (6, 19, 34, 37L and 153), glutamate metabotropic receptor 3, 2 serotonin receptors (1E and 6) and the purinergic receptor Y12.

**FIGURE 5:**
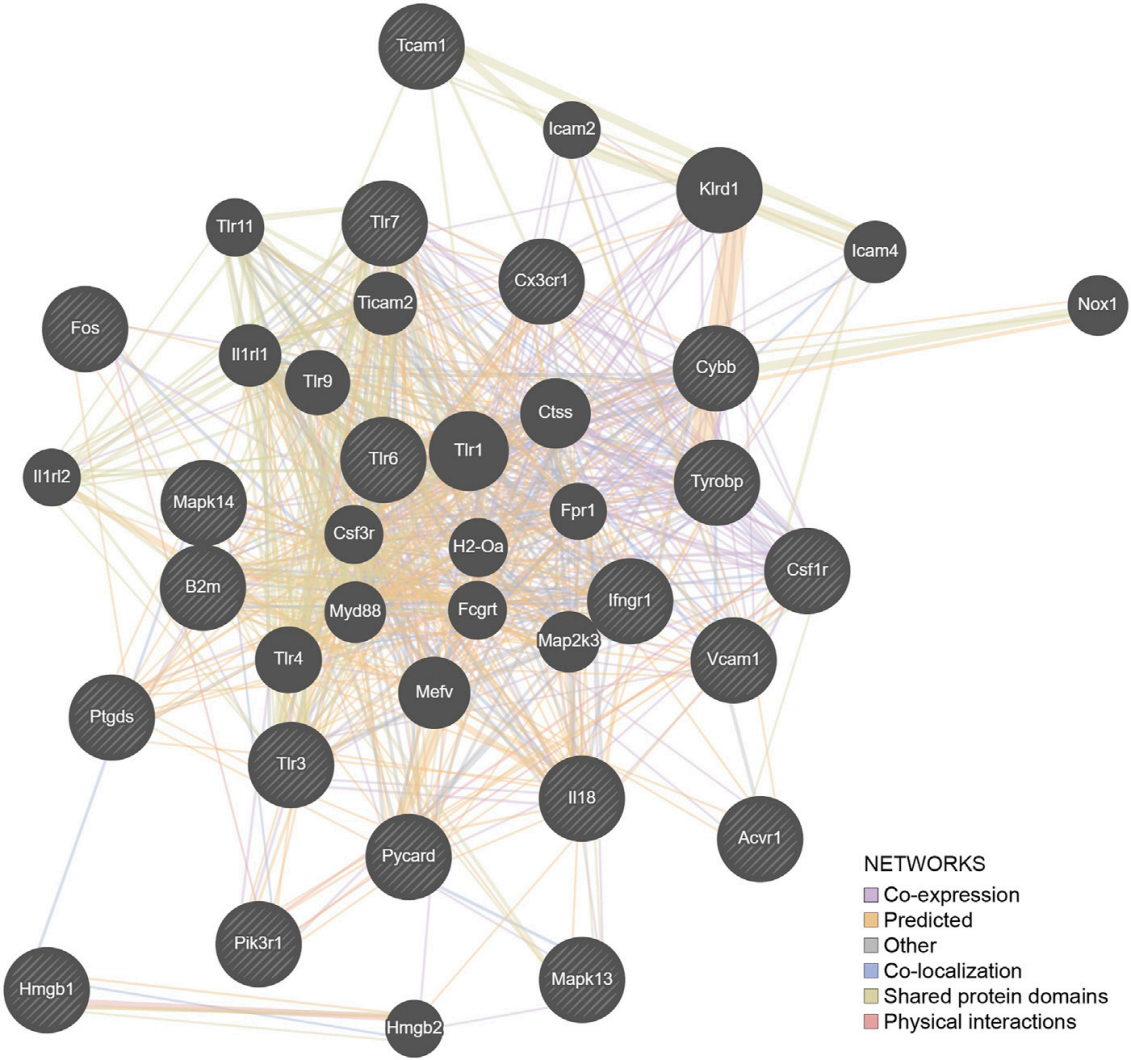
The genes (*N* = 21) detected by IPA as involved in the neuroimmune pathway were entered into a representation using the GeneMANIA algorithm. The hatched genes are these genes. The solid genes are those added by the algorithm to complete the pathways. Fifty percent of the network structure is associated with coexpression, and 20% is associated with predicted associations.

Figure 3B illustrates the marked effects of chronic ethanol consumption and repeated abstinence on the canonical pathways, e.g., the G-Protein Coupled Receptor Signaling pathway is now trending to a positive Z score. Emerging in the HD→VHD group is the neuroinflammation signaling pathway (Z < −2). Genes detected in this pathway included beta-2 microglobulin, interleukin 18, MAP kinases 13 & 14 and Toll-like receptors (3, 6 & 7). Figure 5 illustrates the co-expression patterns of these genes and closely related neuroimmune genes. The IPA generated neuroinflammation pathway is presented schematically in Supplementary Figure S1. It is important to note that the pathway includes elements from microglia, astrocytes and neurons.

Another the top canonical pathways for the HD →. VHD group is RANK signaling in osteoclasts (Z > 2). There is no IPA pathway for brain RANK signaling. However, RANK signaling in the brain is well described [34], where it appears to serve multiple functions in the neuroinflammatory process, e.g., it is neuroprotective in TLR-mediated inflammation. DE genes in this pathway included mitogen-activated protein kinase kinases (3K4, 3K12, 3K13 and 3K14), mitogen-activated protein kinases (13 & 14) and NFKB-inhibitor delta.

## Discussion

Walter et al. [35] were the first to use the within-subject biopsy/necropsy design to examine the effects of chronic open-access ethanol consumption on Area 46 gene expression. That study used cynomolgus macaques (6 controls and 17 drinkers), aged between 5 and 7 years, and the duration of the open-access phase was approximately 28 weeks, with no imposed abstinence. The key findings may be summarized as follows. Compared to the controls, a total of 675 genes were significantly downregulated following EtOH consumption; these genes were functionally enriched for immune response, cell adhesion, plasma membrane, and extracellular matrix. A total of 567 genes were significantly upregulated following EtOH consumption, were enriched in microRNA target sites, and included target sites associated with Toll-like receptor pathways. The differentially expressed genes were also significantly enriched in transcription factor binding sites. Discussion of these data emphasized the effects on the immune response genes, which were both pro- and anti-inflammatory, e.g., *Il1r1* and *Cd74* vs. *Il6r* and *Il10ra*. The overall inflammatory status of area 46 was not known, but the data suggested that in association with chronic ethanol consumption, there was a reset between the pro- and anti-inflammatory pathways. There is robust evidence of relationships between neuroimmune signaling and alcohol use disorder (e.g., [36–38]). Neuroinflammatory mechanisms are associated with both the risk for and individual variation in excessive EtOH consumption (e.g., [39, 40]). Furthermore, there is ample evidence that chronic EtOH consumption has a marked effect on the expression of cytokine ligands and receptors [41–45]. It should be noted that in Walter et al. [35] and the current study, the biopsy/necropsy samples were not perfused. Thus, the possibility must be considered that some of the gene expression signals, especially immune-related signals, are associated with blood cells. For example, Sureshchandra et al. [46] and Barr et al. [47], using gene expression profiling, found that in HD macaques, there was a disruption of innate immunity. However, the profiles of the blood genes affected included an enrichment in genes associated with wound healing and blood coagulation that was not seen in the brain data.

The possibility cannot be easily dismissed that the Area 46, which is part of the cognitive circuit involved in the control of alcohol consumption, does not fit the generally accepted model of the interaction between the neuroimmune system and alcohol consumption. However, we should also note that positron emission (PET) studies using ^18^F-deoxyglucose (FDG) during initial abstinence have revealed in humans that the prefrontal cortex is hypometabolic (Volkow et al. 1992). We assume, although unproven, that similar data would be obtained in the NHP model. It is possible that the neuroimmune genes are particularly sensitive to this metabolic malaise. The duration of the metabolic effect under the conditions of the present experiment is unknown.

The current study used the within-subject biopsy necropsy design, along with repeated cycles of abstinence, to examine transcriptional changes associated with ethanol intake and ADE. The study differs from Walter et al. [35] in several important aspects. 1) Male rhesus macaques rather than cynomolgus macaques were the study subjects; however, the age range was similar. 2) The initial open-access ethanol drinking phase was significantly longer (60 weeks vs. 28 weeks). An additional 52 weeks of open-access consumption (two post-abstinence open access periods of approximately 26 weeks each). 3) The necropsy sample was obtained after 34–38 days of withdrawal. Despite these differences, both studies detected an “immune response” signal. As noted above, in Walter et al. [35], the signal was detected in genes downregulated in drinkers but not controls. In the current study, the signal was detected in those subjects where the ADE moved the HD group to VHD status. The question naturally arises as to whether the immune response genes were similar. This is a hypothetical question that cannot be addressed satisfactorily given the differences in study design and given that the number of immune response genes detected in Walter et al. [35] was markedly larger (92 vs. 34) than the number detected in the current study. However, 13 of the genes were in common: *C3*, *Csf1r*, *Ctss*, *Fgr*, *Ifi27*, *Il16*, *Irf8*, *Lyn*, *Ncf2*, *Pycard*, *Tyrobp*, *Vav1* and *Was*. With the exception of Lyn tyrosine kinase (*Lyn*), this core gene grouping would be considered proinflammatory. The colony stimulating factor 1 receptor (CSF1R) and its ligand (CSF1) are key to the maintenance and proliferation of microglia, the resident macrophages in the brain. Given the role(s) of immune systems in excessive ethanol consumption noted above, microglia are likely to have some role in this process. Fortunately, this hypothesis can be directly tested since CSF1R antagonists deplete brain microglia. Warden et al. [48, 49] examined this issue from two perspectives. They found that PLX5622-induced depletion of microglia did not affect the hypnotic or sedative effects of acute ethanol intoxication and did not affect the maintenance or escalation of voluntary ethanol consumption. However, microglial depletion blocked the poly (I:C)-induced escalation of ethanol consumption, suggesting that microglia have a role in ethanol consumption in the context of sufficient immune activation [48]. When they used PLX5622 to examine the role of microglia in chronic intermittent ethanol (CIE) vapor-induced escalation of ethanol consumption (2-bottle choice), microglia depletion blocked CIE-induced increases in ethanol consumption and blocked associated CIE-induced changes in gene expression [49]. The question naturally arises as to how to interpret the decrease in *Csf1r* expression observed in Walter et al. [35] and the current study. One can speculate that in macaques, the decreased expression is part of a compensatory mechanism, perhaps involving all of the genes noted above, in response to high levels of ethanol consumption.

Continuing the theme of compensatory mechanisms is the observation that the RANK signaling pathway was significantly upregulated in the necropsy vs. biopsy samples of the subjects that converted from HD to VHD. Key protein members of the pathway include receptor activator of nuclear factor-κB (RANK), the RANK ligand (RANKL), and osteoprotegerin (OPG), forming the RRO axis. OPG is a decoy receptor for RANKL. The involvement of the RRO axis in bone remodeling and peripheral immune responses is well established [50–53]. In the brain, the RRO axis tunes the neuroinflammatory response, depending on the molecular, cellular and pathological context. RANK/RANKL signaling is neuroprotective in TLR-mediated inflammation, which has been linked to ethanol-induced neuroinflammation (see, e.g., [54–57]). To our knowledge, this is the first report linking RANK signaling to excessive ethanol consumption. Given the large number of compounds and biologics (e.g., antibodies) that have been developed to affect bone remodeling via RANK signaling, some of these may be effective CNS therapies [34]. However, we also consider that the RANK signaling changes may be protective adaptations and not especially suitable as therapeutic targets. A similar argument could be raised for the changes in MAPK signaling (see below).

Given the widespread association of MAPK signaling with numerous brain mechanisms and pathways, it cannot be surprising to have detected an association between MAPK signaling and very heavy drinking. From a genomics perspective, Mulligan et al. [58], in a meta-analysis of microarray data, detected an association between MAPK signaling and ethanol preference. Subsequent studies (e.g., [59, 60]) have confirmed the association between Ras/MAPK pathways and preference consumption. Here, we focus on Ferguson et al. [10], who examined and reviewed 17 human postmortem studies involving a total of 230 cases and 233 controls; the data were derived from both microarray and RNAseq experiments and involved multiple brain regions. Upregulated KEGG pathways in cases included focal adhesion, MAPK signaling, cytokine-cytokine receptor interaction, cell adhesion and regulation of actin cytoskeleton. Downregulated KEGG pathways included oxidative phosphorylation, Parkinson’s disease and DNA replication. (See Table 2—Ferguson et al. [10]—for a complete list of up- and downregulated KEGG pathways). In the current study, we observed that VHD is associated with the upregulation of genes that negatively impact MAPK signaling. The signal was seen in both the HD→VHD and the VHD→VHD groups but not in the BD→HD group. Furthermore, the MAPK signal was not detected in Walter et al. [35], although the downregulation of immune-related genes was observed (see above). Overall, these data suggest that ADE leads to MAPK signaling. The question we cannot answer is whether there was an upregulation of MAPK genes [10] that preceded the upregulation of genes that would blunt such an effect. Is this another example of compensation? It is of interest that a core set of genes associated with the negative regulation of MAPK signaling was the same in the HD→VHD and VHD→VHD groups. The core set included dual specificity phosphatases, members of the sprouty/spred gene family, *Rgs2* and *Bmp4*. Here, we note that the DUSP and SPRY/SPRED proteins have been suggested to be novel targets for CNS therapeutics [61, 62].

The current results provide new insights into the transcriptional changes that occur over the period from late adolescence to early adulthood (roughly 8–10 macaque years) and provide new information on how chronic ethanol consumption affects these transcriptional changes. Detailed information on the spatiotemporal dynamics of the developing NHP brain transcriptome is available (e.g., [63, 64]). However, these studies do not cover the period examined in the current study. The data presented here are clear that there are marked transcriptional changes that occur over young adulthood. The main caveat to these data is the procedures the animals encountered over the nearly 3-year duration of the study. The animals were anesthetized and underwent surgery. The pre-alcohol biopsy sample was removed in all but four low to binge drinkers, maltodextrin was administered chronically, and the animals were individually housed. The precise impact of these manipulations and circumstances are unknown. However, we observed significant downregulation of genes associated with cell migration, signal transduction and the plasma membrane in the control animals in biopsy compared to necropsy samples. Upregulated genes were associated with translation. In the BD→HD group, these ontologies were reduced, further reduced in the HD→VHD group and absent in the VHD→VHD group. Assuming that the transcriptional changes seen in the controls are at least, in part, signatures from late cortical developmental processes, ethanol plus the ADE reverses these changes in a dose-related fashion. What is not known is the persistence of the ethanol effects. That is, the data do not address if the absence of altered gene expression ontologies seen in ethanol drinkers compared to controls is representative of a “critical window” such that brain growth trajectories will not recover.

Given our understanding of the mechanisms associated with chronic ethanol intoxication and withdrawal in highly dependent individuals, the data presented here suggest that in the NHP brain, area 46 has relatively subtle adaptations, perhaps reflecting the relative homogeneity of the prefrontal molecular/granular layers examined here. The question then arises: do other brain regions drive excessive consumption? Previous studies (e.g., [65]) have suggested that the central nucleus of the amygdala (CeA) is key in regulating ethanol preference. Iancu et al. [11] aligned gene expression in Area 32 and the CeA with the drinking patterns in 31 rhesus macaques that had completed the standard 12-month open-access ethanol consumption protocol [20]. The CeA transcriptional data, compared to the cortical Area 32 data, aligned more significantly with the drinking pattern data. Membrane and synaptic genes, e.g., *Chrm3*, *Chrna4*, *Chrna7*, *Glra2*, *Grm1* and *Grm2*, were especially enriched in the genes positively correlated with ethanol consumption. This signal was not observed in Area 46. Overall, the data presented here are part of a larger and ongoing effort [9, 11, 35, 66] to understand how chronic ethanol consumption affects the primate brain, including expanding the analyses to both males and females.

The data presented here have translational value for understanding the effects of voluntary chronic ethanol drinking, coupled with periods of involuntary abstinence, on subsequent drinking patterns. The data illustrate that across all levels of NHP ethanol consumption, imposed abstinence increased consumption when ethanol was reintroduced. The approximately 4 weeks of abstinence was modeled after the most common inpatient treatment protocol for severe AUD [8]. Escalated drinking, as seen in the return to heavy drinking following imposed abstinence, is a hallmark symptom of the addiction cycle [67] and is commonly modeled in rodents [68]. Furthermore, for many monkeys, the effects of repeated abstinence on ethanol intake escalated to higher steady state levels, for example, in the conversion of HDs to VHDs and the increase in consumption among the VHDs. As we hypothesize that the escalated drinking after abstinence cycles represent a model of the most severe AUD cases, we focused on the transcriptional effects of repeated abstinence to suggest or support new strategies for pharmacotherapeutic development that could be incorporated into abstinence-based therapies. As noted above, the within-subject DLPFC Area 46 transcriptional changes suggest that neuroinflammation mechanisms are prominent in the state of the brain about to return to heavy drinking (i.e., in prolonged abstinence just prior to resumed access to alcohol). Although neuroinflammation may be protective in some situations [69], recent data suggest that centrally acting anti-inflammatory drugs, such as the PDE4 inhibitor apremilast, could be beneficial in the treatment of AUD [70]. The data presented here suggest that drugs that affect MAPK and RANK signaling may also be effective, especially in therapeutic situations associated with involuntary abstinence.

## Data Availability

The raw fastq files for this dataset are available for download at NIH's Sequence Read Archive (https://www.ncbi.nlm.nih.gov/bioproject/PRJNA1050227/) under accession PRJNA1050227.

## Footnotes

1
MATRR.com

2
http://www.bioinformatics.babraham.ac.uk/projects/fastqc/

3
http://multiqc.info/

4
https://www.R-project.org

5
https://www.qiagenbioinformatics.com/products/ingenuity-pathway-analysis
