# Supplementary figures and images for: Effects of repeated alcohol abstinence on within-subject prefrontal cortical gene expression in rhesus macaques
Source: Adv Drug Alcohol Res. 2024 Apr 26;4:12528. doi: 10.3389/adar.2024.12528 (PMC11082748; doi:10.3389/adar.2024.12528)

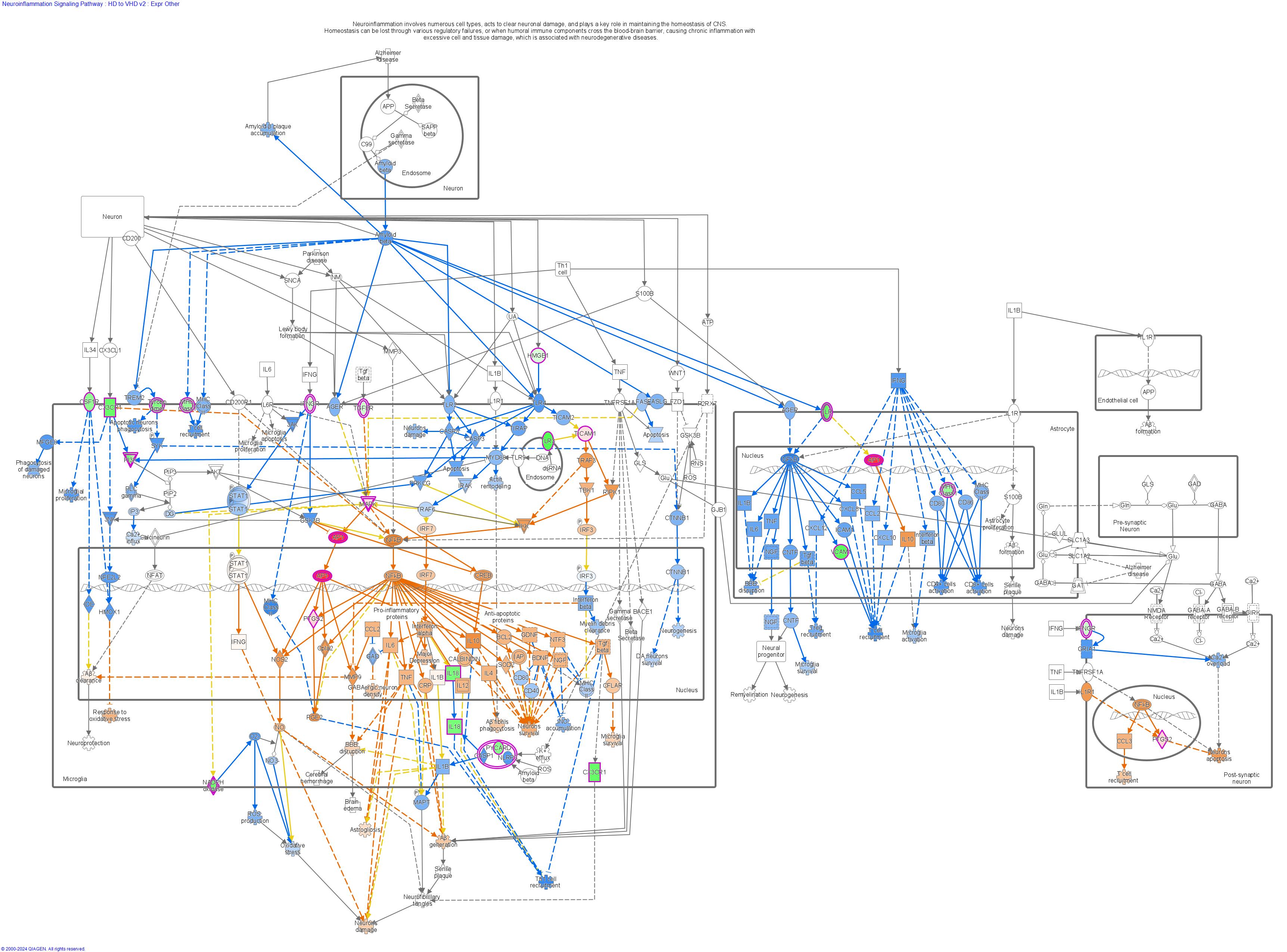

Supplement: Supplementary file 2 [file Image1.JPEG]
