# Supplementary material for: Effects of repeated alcohol abstinence on within-subject prefrontal cortical gene expression in rhesus macaques
Source: Adv Drug Alcohol Res. 2024 Apr 26;4:12528. doi: 10.3389/adar.2024.12528 (PMC11082748; doi:10.3389/adar.2024.12528)

Cohort 10 and 14 RNA-seq DE analysis, v9

BBC

2024-02-27

Contents

Notes 2

EDA 2

Design table inspection . . . 2

Filtering and Normalization . . . 3

Box plots and density plots of normalized log-CPM values across samples . . . 4

By group . . . 4

By Timepoint . . . 6

By drGroup_open1 . . . 7

By drGroup_open3 . . . 8

By Cohort . . . 9

Unsupervised sample clustering . . . 10

Unsupervised sample clustering at each Timepoint with Cohort effect removed . . . 12

Differential expression analysis 15

`DRINKERvsCONTROL_bio` . . . 17

`HDVHDvsLDBD_bio` . . . 17

`VHDvsHD_bio` . . . 18

`DRINKERvsCONTROL_nec` . . . 18

`VHDvsHD_nec` . . . 19

`control_nec.vs.bio` . . . 19

`HD.to.VHD_nec.vs.bio` . . . 19

`VHD.to.VHD_nec.vs.bio` . . . 20

`BD.to.HD_nec.vs.bio` . . . 20

`DRINKER_change_vs_CONTROL_change` . . . 20

Summary of DE results and plots 20

Notes

- Cohort 10 and 14 biopsy and pre-perfusion necropsy area 46 samples, all male
- Stringent low count filtering to keep less genes for DE analysis, as instructed by investigators
- Sample table used is `Coh10_Coh14_drinkingcategory_biopsy.xlsx` received on 11/19/2021 from Natali.
- Investigators specified the comparisons to be made.
- 2/4/2024, specified contrasts requested by reviewers added.

EDA

Design table inspection

Table 1: RNA-seq libraries

MATRRID	Cohort	Group	bio	nec
10210	10	ethanol	S10210_A12_R_bio_pre	S10210_A12_L_nec_pre
10211	10	ethanol	S10211_A12_L_bio_pre	S10211_A12_R_nec_pre
10212	10	ethanol	S10212_A12_L_bio_pre	S10212_A12_R_nec_pre
10215	10	ethanol	S10215_A12_R_bio_pre	S10215_A12_L_nec_pre
10208	10	ethanol	NA	S10208_A12_R_nec_pre
10209	10	ethanol	NA	S10209_A12_R_nec_pre
10213	10	ethanol	NA	S10213_A12_R_nec_pre
10214	10	ethanol	NA	S10214_A12_L_nec_pre
10220	10	control	S10220_A12_R_bio_pre	S10220_A12_L_nec_pre
10221	10	control	S10221_A12_L_bio_pre	S10221_A12_R_nec_pre
10222	10	control	S10222_A12_R_bio_pre	S10222_A12_L_nec_pre
10223	10	control	S10223_A12_L_bio_pre	S10223_A12_R_nec_pre
10242	14	ethanol	S10242_A46_biop_pre_etoh	S10242_A46_biop_post_etoh
10243	14	ethanol	S10243_A46_biop_pre_etoh	S10243_A46_biop_post_etoh
10244	14	ethanol	S10244_A46_biop_pre_etoh	S10244_A46_biop_post_etoh
10246	14	ethanol	S10246_A46_biop_pre_etoh	S10246_A46_biop_post_etoh
10247	14	ethanol	S10247_A46_biop_pre_etoh	S10247_A46_biop_post_etoh
10248	14	ethanol	S10248_A46_biop_pre_etoh	S10248_A46_biop_post_etoh
10249	14	ethanol	S10249_A46_biop_pre_etoh	S10249_A46_biop_post_etoh
10251	14	ethanol	S10251_A46_biop_pre_etoh	S10251_A46_biop_post_etoh
10252	14	ethanol	S10252_A46_biop_pre_etoh	S10252_A46_biop_post_etoh
10241	14	control	S10241_A46_biop_pre_etoh	S10241_A46_biop_post_etoh
10245	14	control	S10245_A46_biop_pre_etoh	S10245_A46_biop_post_etoh
10250	14	control	S10250_A46_biop_pre_etoh	S10250_A46_biop_post_etoh

- 44 RNA-seq libraries from matched biopsy and necropsy samples originated from 24 animals. 4 animals from cohort 10 do not have biopsy samples available.

Table 2: drinker category at open access 1 and 3

drGroup_open1	drGroup_open3	n
LD	LD	1
LD	BD	1

drGroup_open1	drGroup_open3	n
LD	HD	1
BD	BD	1
BD	HD	4
HD	VHD	4
VHD	VHD	5

Filtering and Normalization

Suzi Fei processed the raw RNA-seq data (Please refer to Suzi's report for details) and passed to us summarized raw counts of 30683 genes from 44 libraries.

It has been shown that genes with very low counts across all samples provide little evidence for differential expression. Also, the pronounced discreteness of these very low counts impairs validity and effectiveness of down stream statistical DE analysis. We therefore filter out these extremely low count genes before further analysis. Here "extreme low counts" is defined as no more than 2 count-per-million (CPM) in a sample, which corresponds to 23 - 24 count in the smallest library (Usually a gene is required to have a count of 5-10 in a library to be considered expressed in that library). To retain genes that are only expressed in the smallest group (which can be strong evidence for DE under experimental treatments), we filter to keep genes that pass extreme low counts criteria in at least 7 samples. 13868 genes are retained after extreme low count filtering.

Under the assumptions that all samples should have a similar range and distribution of expression values, and that there is roughly symmetric differential expression, we normalized raw counts with TMM method (trimmed mean of M values).

Box plots and density plots of normalized log-CPM values across samples

By group

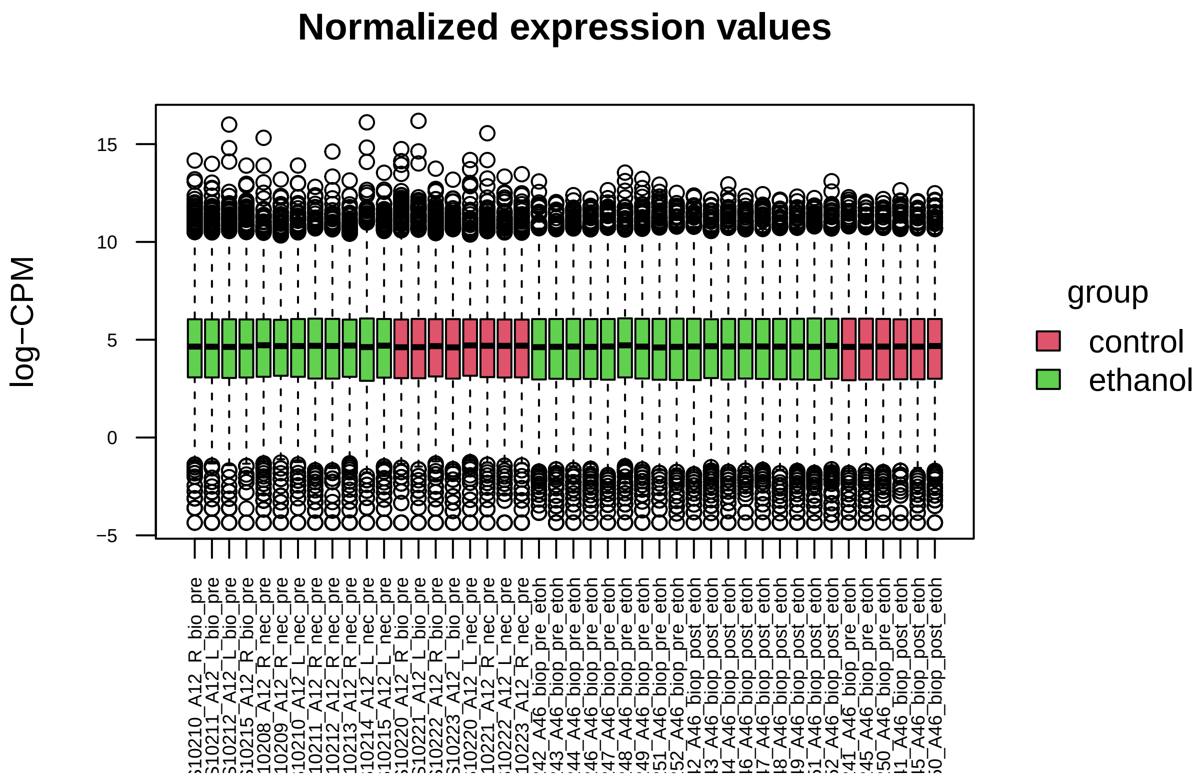

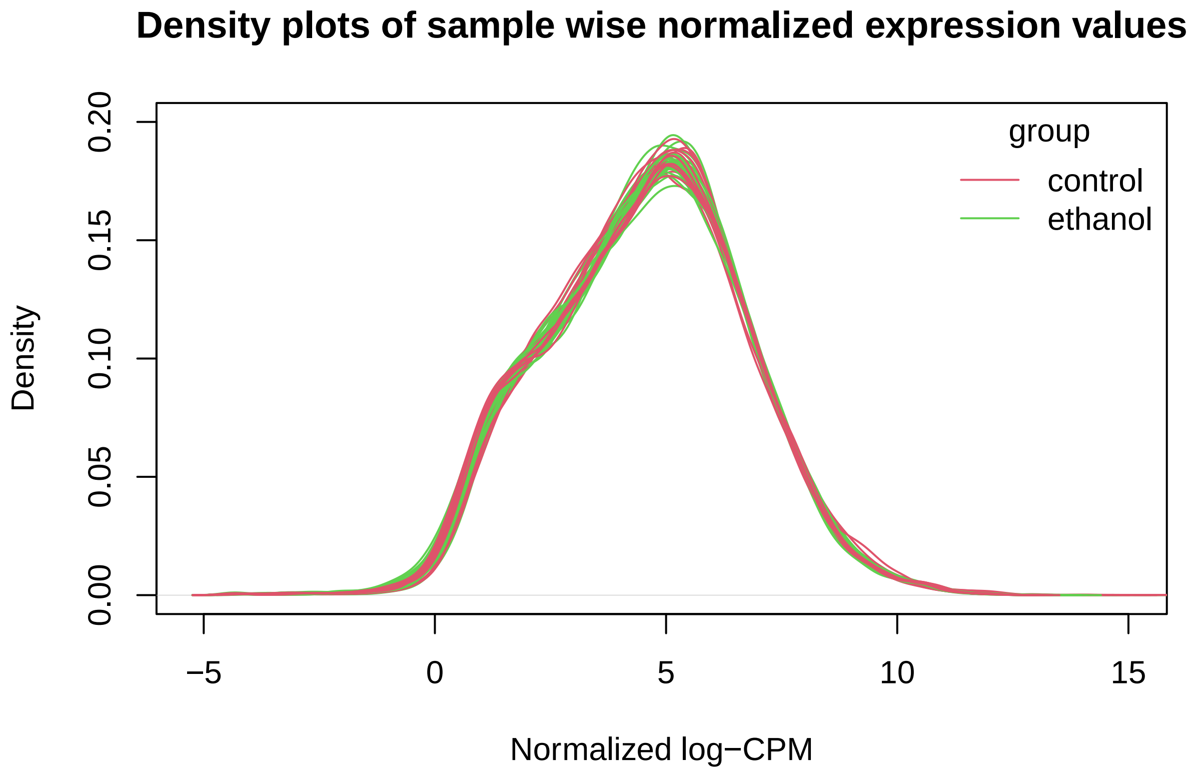

Density plots of sample wise normalized expression values
group
control
ethanol
Density
0.00
0.05
0.10
0.15
0.20
−5
0
5
10
15
Normalized log−CPM

By Timepoint

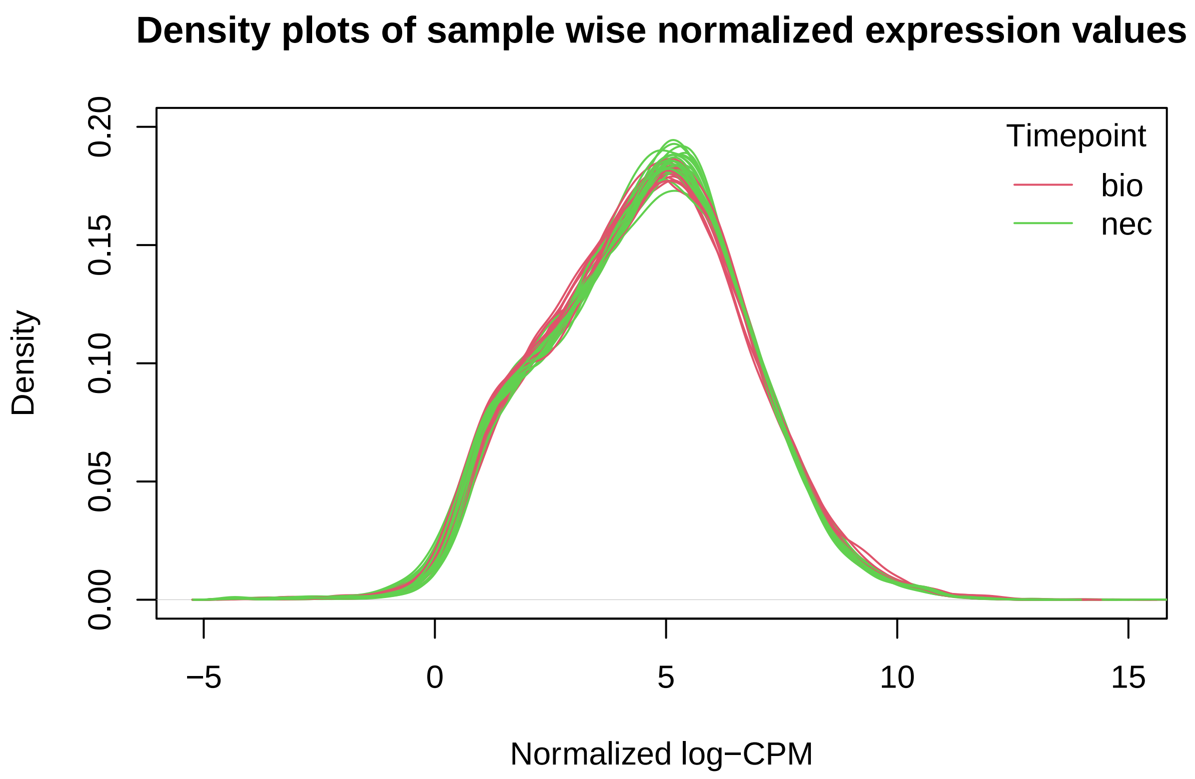

By drGroup_open1

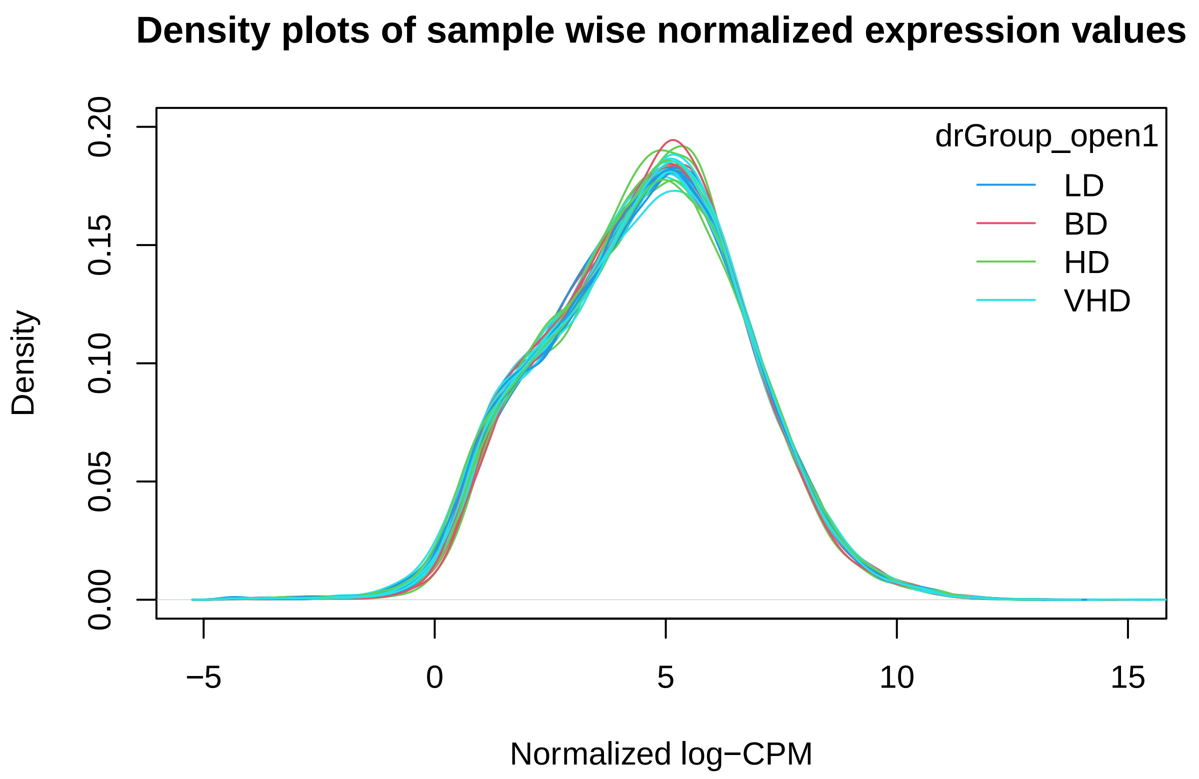

By drGroup__open3

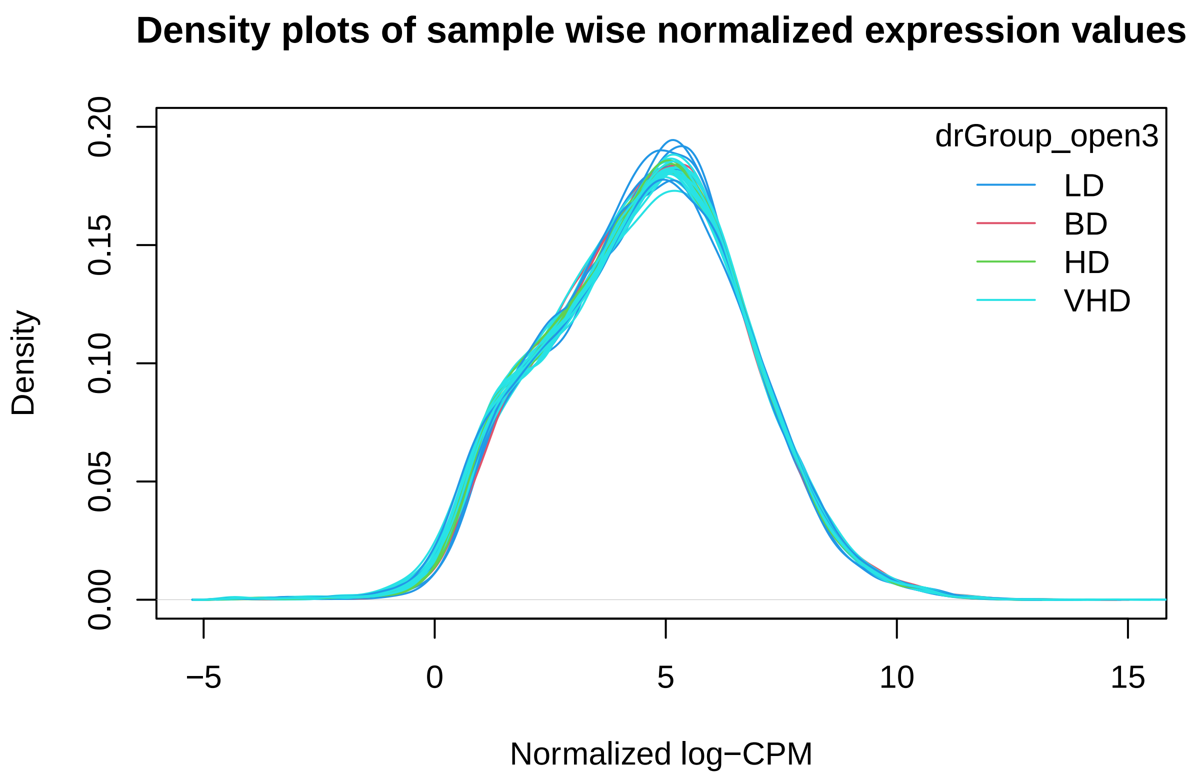

By Cohort

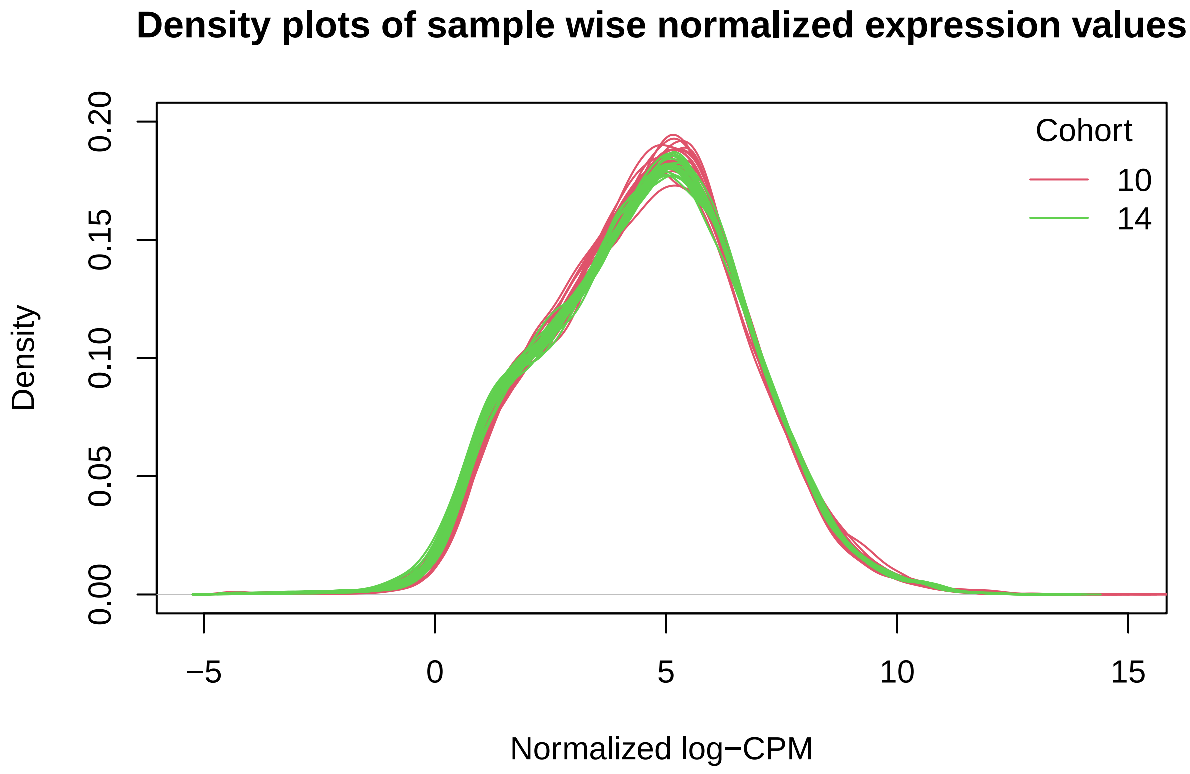

- After normalization, most samples seem to show similar global distribution in sample wise expression.
- Largest variation may come from Cohort and Timepoint.

Unsupervised sample clustering

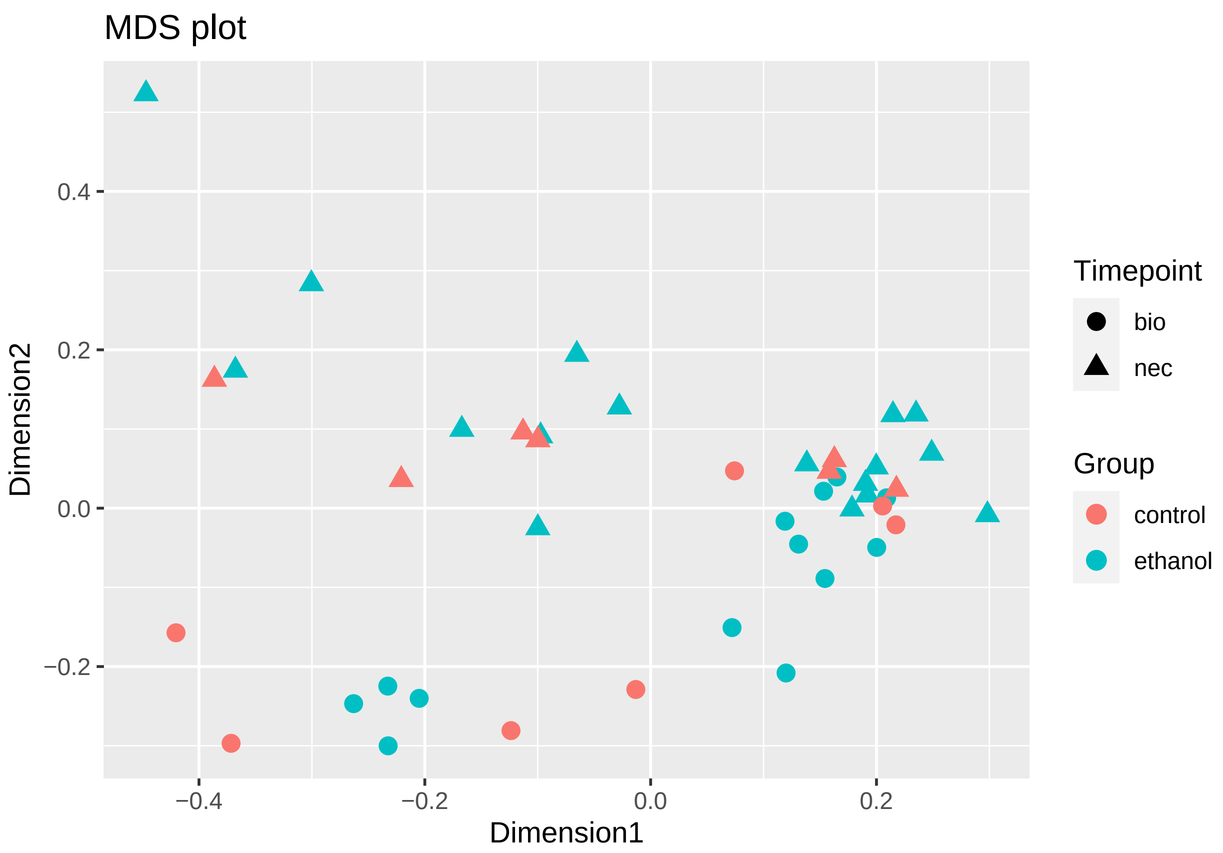

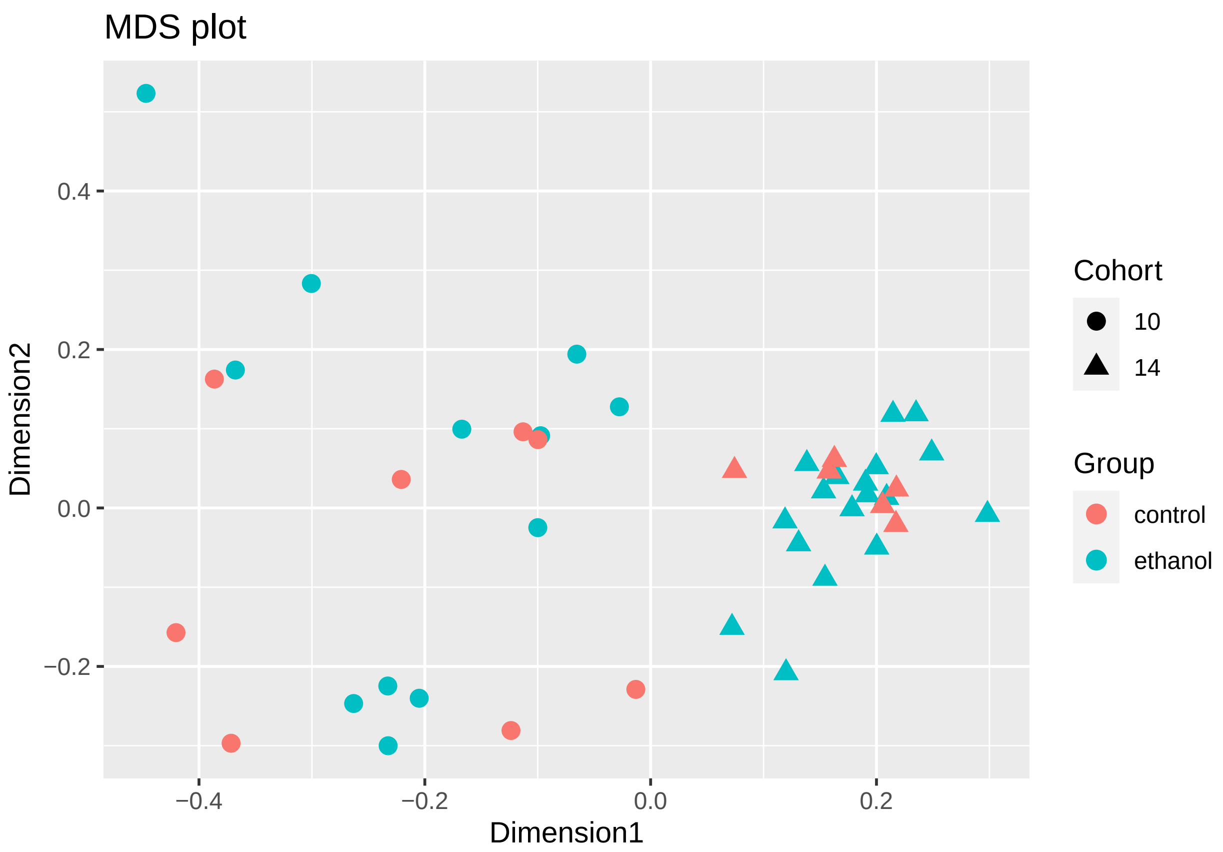

Here we used multidimensional scaling (MDS) plots to show similarities and dissimilarities between samples in an unsupervised manner; therefore sample clustering in MDS plots can help identify factors that contribute to variation in gene expression.

- We see again major variation may come from Cohort and Timepoint. We'll adjust for Cohort in the model.
- Next we'll look at sample clustering with cohort effect removed at each timepoint. The goal is to visualize within drinkers how different the categories are from each other.

Unsupervised sample clustering at each Timepoint with Cohort effect removed

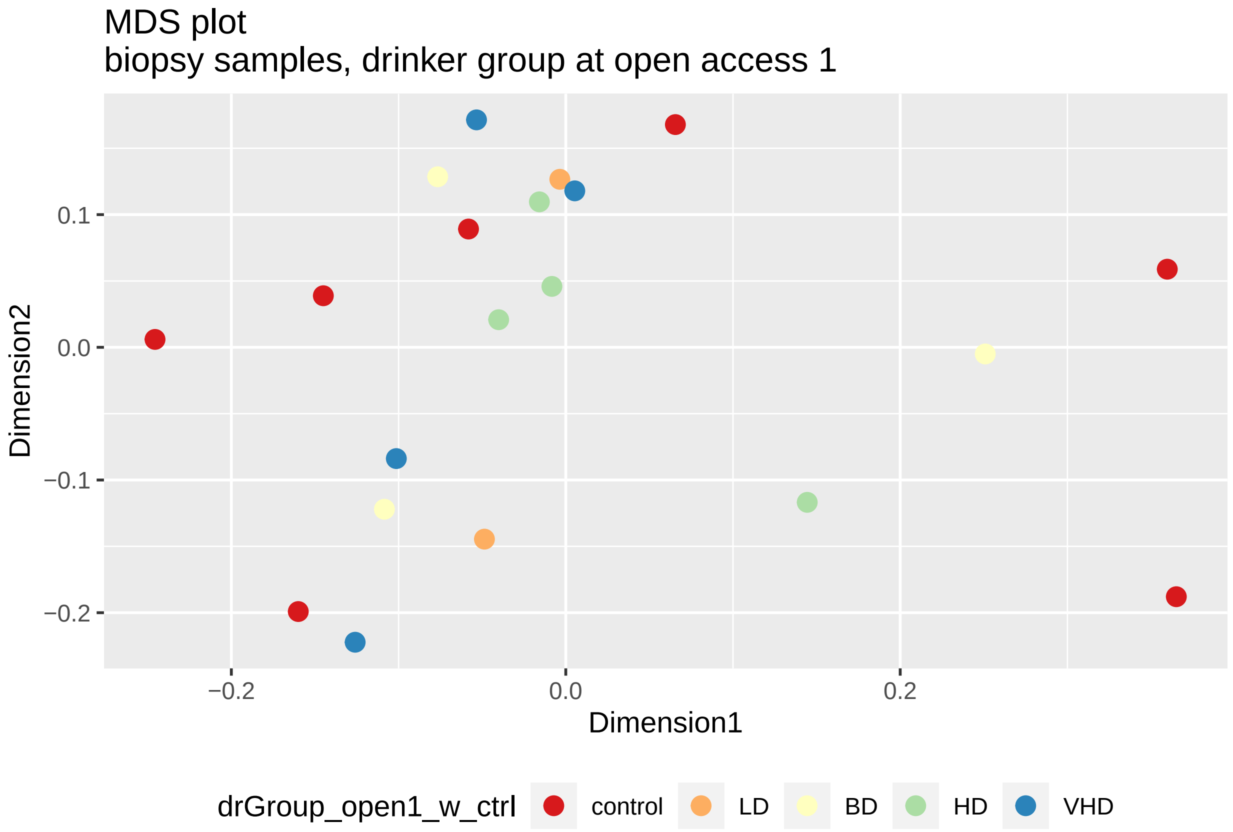

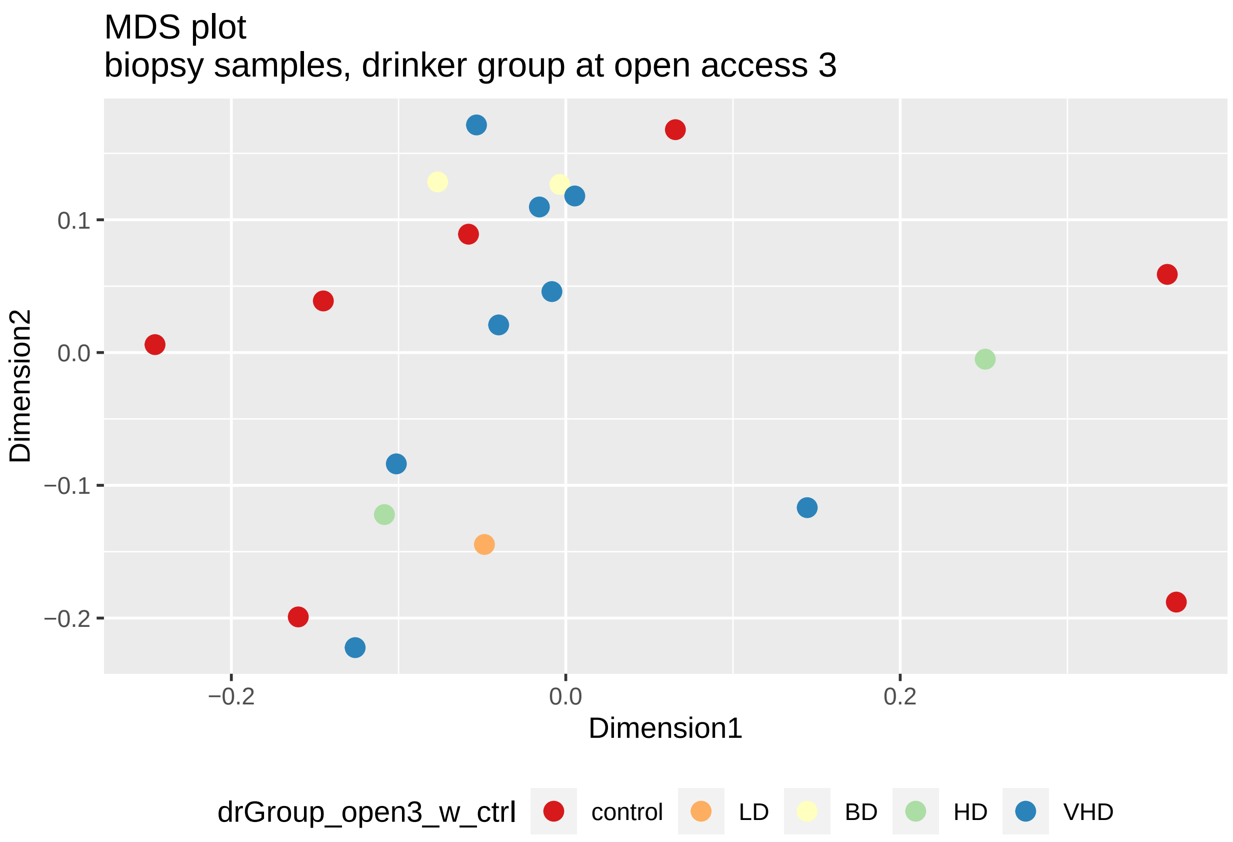
MDS plot
biopsy samples, drinker group at open access 3
Dimension2
0.1
0.0
−0.1
−0.2
−0.2
0.0
0.2
Dimension1
drGroup_open3_w_ctrl
control
LD
BD
HD
VHD

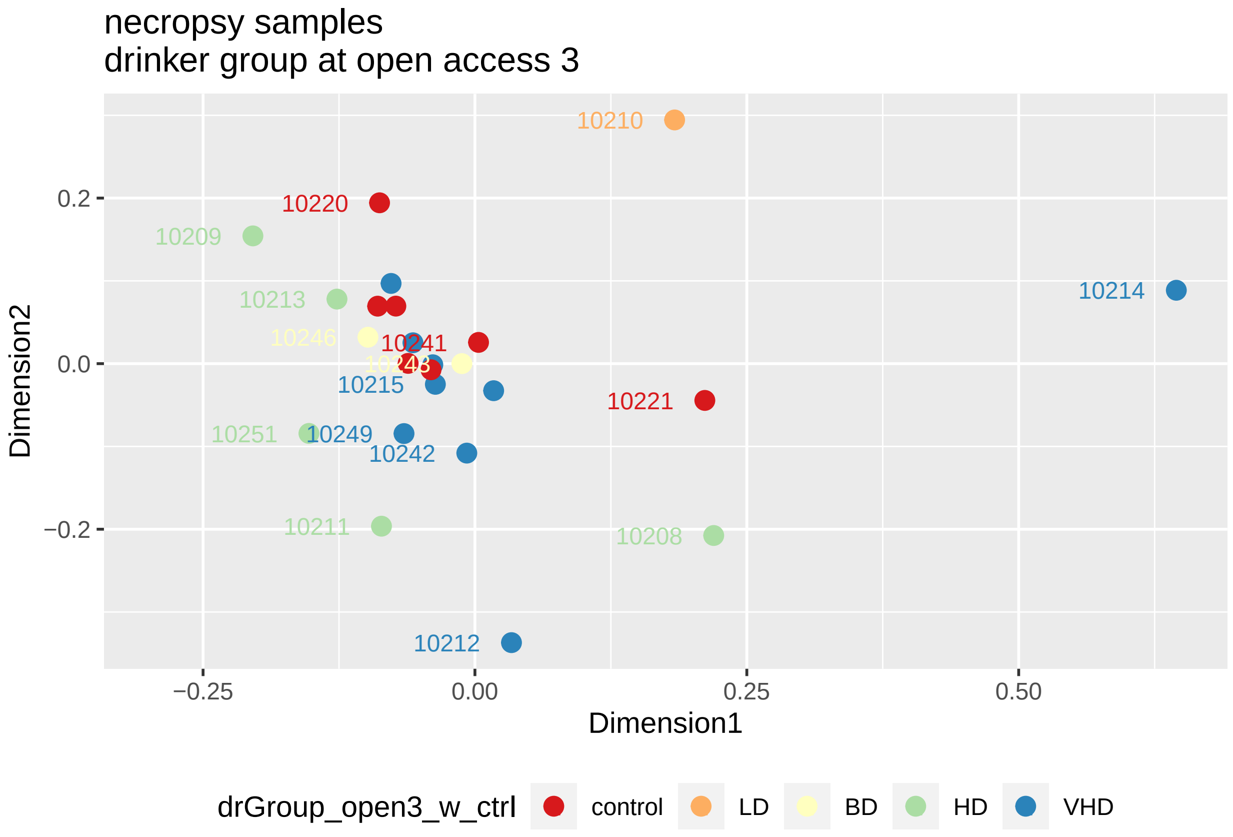
necropsy samples
drinker group at open access 3
10210
10220
10209
10214
10213
10246
10241
10243
10215
10221
10251
10249
10242
10211
10208
10212
0.2
0.0
−0.2
−0.25
0.00
0.25
0.50
Dimension1
Dimension2
drGroup_open3_w_ctrl
control
LD
BD
HD
VHD

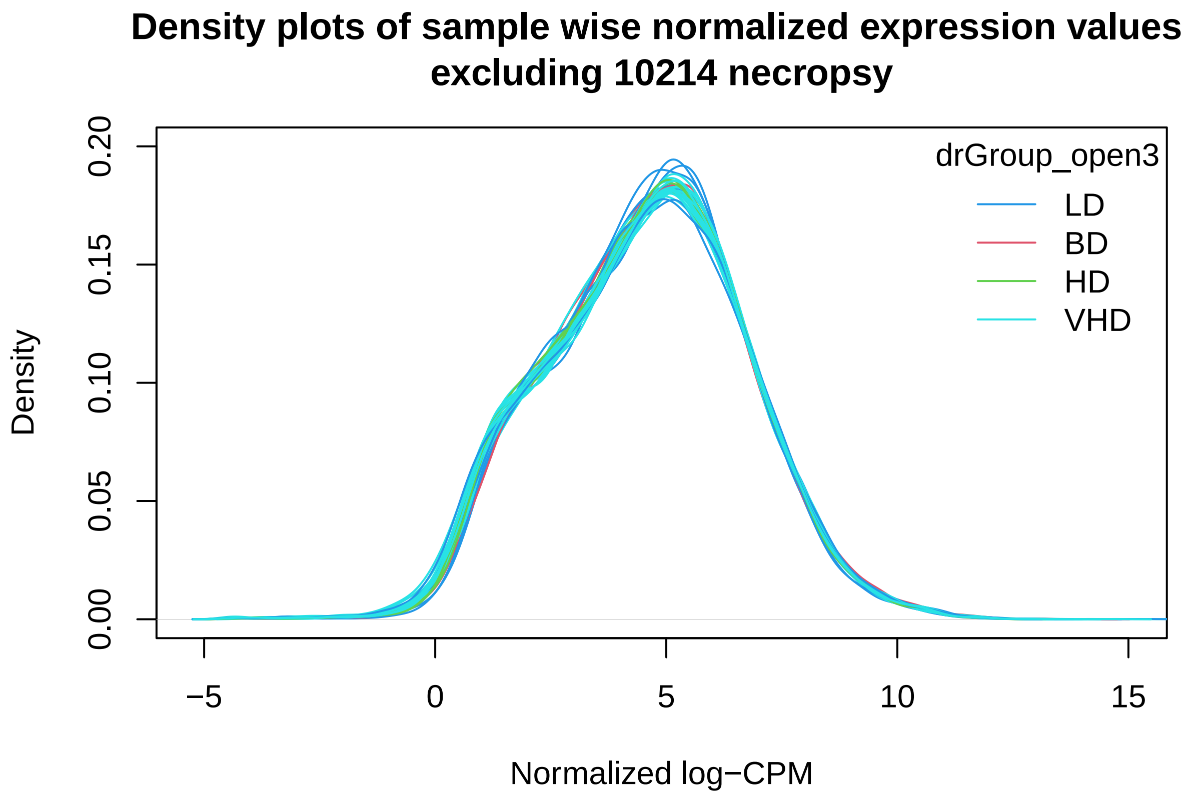

- Biopsy samples don't seem to cluster by drinker category at open access 1 or open access 3.
- In necropsy samples, HD and VHD seem to have similar expression profile and some animals in these two groups seem to be different from control animals. LD and BD group have too few samples to see any pattern.
- In necropsy samples, one VHD (MATRRID 10214) seems to be different from the rest of VHDs. This animal is one of those that don't have biopsy sample. This sample is indeed the one sample that shows slightly different global distribution in density plot. We therefore excluded this sample from analysis.
- The following DE analysis include 43 samples

Differential expression analysis

For DE analysis, we will use `limma voom` workflow. Briefly, The `voom` transformed expression values carrying observational level precision weights were put into `limma` empirical Bayes analysis pipeline to identify DE genes. Common to most gene expression studies, small sample size makes estimation of variation unstable. The empirical Bayes approach first fit gene-wise linear models, followed by robust empirical Bayes moderation of the estimated sample variances by borrowing information across all the genes to obtain more stable estimates of gene-wise variability, which gives far more stable inference when the sample size is small. In comparisons involving biopsy and necropsy samples from the same subjects, gene-wise within subject correlation was estimated using REML method, then the trimmed mean of atanh-transformed (Fisher's Z transformation) estimated inter-subject correlation was back transformed (tanh transformed) to be used as consensus correlation for all genes. This will given more reliable inference as we have the same issue of small sample size when estimating within-subject correlation.

- Separate models focusing on the difference in expression between groups before drinking started (biopsy samples): With gene-wise linear models on os1 drinking category (including control group), adjusting

for Cohort and surrogate variables identified through SVA, the following between group comparisons were performed. Note that this approach compares the weighted mean between groups. Take the HDVHDvsLDBD comparison as example, all HD and VHD samples are grouped together as one HDVHD group, and all LD and BD samples are grouped together as one LDBD group. Mean in HDVHD group is calculated from HD and VHD groups in proportion to sample size in each group, thus represents weighted mean.

  - DRINKERvsCONTROL_bio: DRINKER - CONTROL
  - HDVHDvsLDBD_bio: HDVHD - LDBD (os1 drinking category)
  - VHDvsHD_bio: VHD - HD (os1 drinking category)

- **Added for paper revision 2/4/2024 and 2/15/2024**: Separate models focusing on the difference in expression between specified groups at necropsy (necropsy samples): With gene-wise linear models on experiment group , adjusting for Cohort and surrogate variables identified through SVA:

  - DRINKERvsCONTROL_nec: DRINKER - CONTROL
  - VHDvsHDL_nec: VHD - HD (os3 drinking category)

- Separate models focusing on the change in expression from biopsy to necropsy in each of the following specified groups: All models contain Timepoint (biopsy/necropsy), adjust for Cohort and account for correlation within animals. Note that SVA is not performed for these comparisons, because it has not been shown that sva performs as well with correlated data). As mentioned above, this set of comparisons involve estimating within-subject correlation. Because each of the following comparisons are done using corresponding subset of samples, within-subject correlation estimates may not be reliable. In fact, we saw in two of the comparisons, consensus within-subject correlation was estimated to be very small or even negative, which was likely not the truth. We therefore decided to use all samples to estimate consensus within-subject correlation, which will be used for each of the comparisons below.

  - control_nec.vs.bio: necropsy - biopsy in control
  - HD.to.VHD_nec.vs.bio: necropsy - biopsy in animals with HD at os1 and VHD at os3
  - VHD.to.VHD_nec.vs.bio: necropsy - biopsy in animals with VHD at os1 and VHD at os3
  - BD.to.HD_nec.vs.bio: necropsy - biopsy in animals with BD at os1 and HD at os3

- **Added for paper revision 2/4/2024**: Using drinker and control group biopsy and necropsy samples, with gene-wise linear models adjusting for Cohort and accounting for correlation within animals (all sample consensus within-subject correlation used, as explained above), hypothesis test for contrast:

  - DRINKER_change_vs_CONTROL_change: (DRINKER_nec-DRINKER_bio)-(CONTROL_nec-CONTROL_bio)

All results were summarized in the accompanying DEresults_v9.csv file, which can be opened in Excel for filtering and sorting. For each contrast, we provide the following metrics:

- log2FC: Fold change of gene expression in log2 scale. The positive values of log2FC for group1-group2 indicate evidence in favor of group1 > group2 (i.e. genes up-regulated in group1), while the negative values indicate evidence in favor of group1 < group2 (i.e. genes down-regulated in group1). For example, log2FC of 1 corresponds to fold change of 2 (up-regulated) and log2FC of -1 corresponds to fold change of 0.5 (down-regulated).
- pvalue: Empirical Bayes moderated p-value for that particular contrast.
- FDRp: FDR (Benjamini and Hochberg, 1995) adjusted p-values. Multiple testing is adjusted down the genes for each contrast separately.

Along with hypothesis testing results, raw counts and normalized counts in CPM are also provided in the result table.

`DRINKERvsCONTROL_bio`

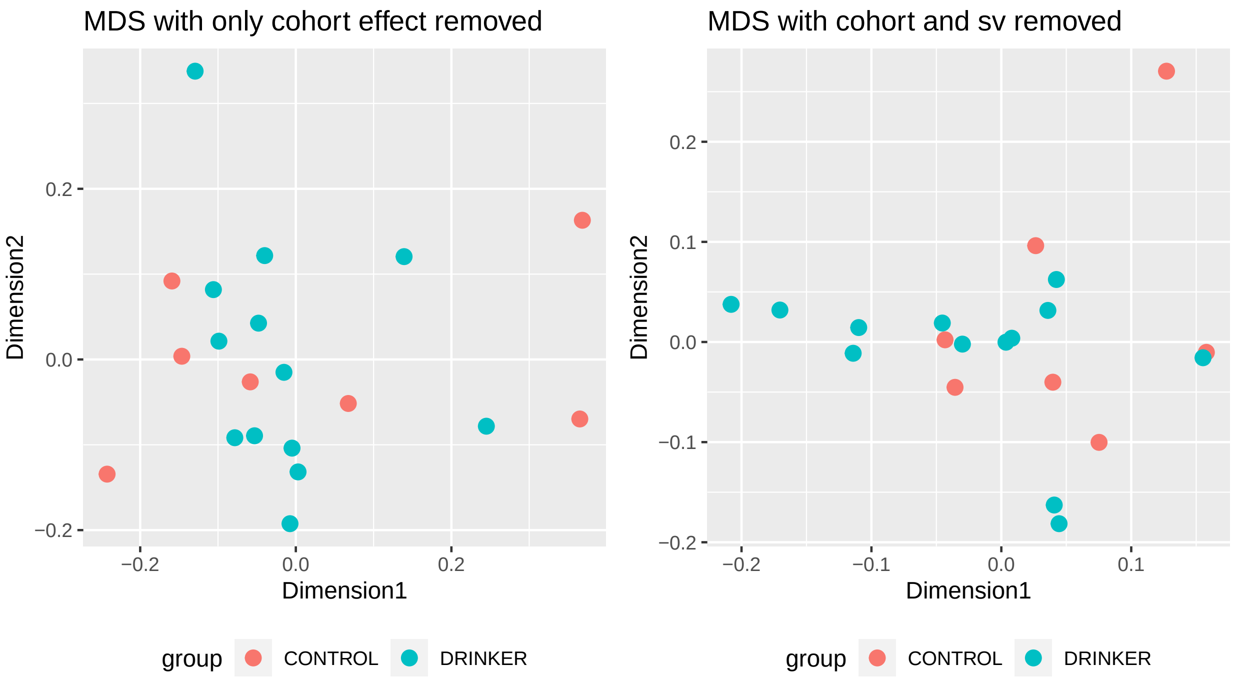

`HDVHDvsLDBD_bio`

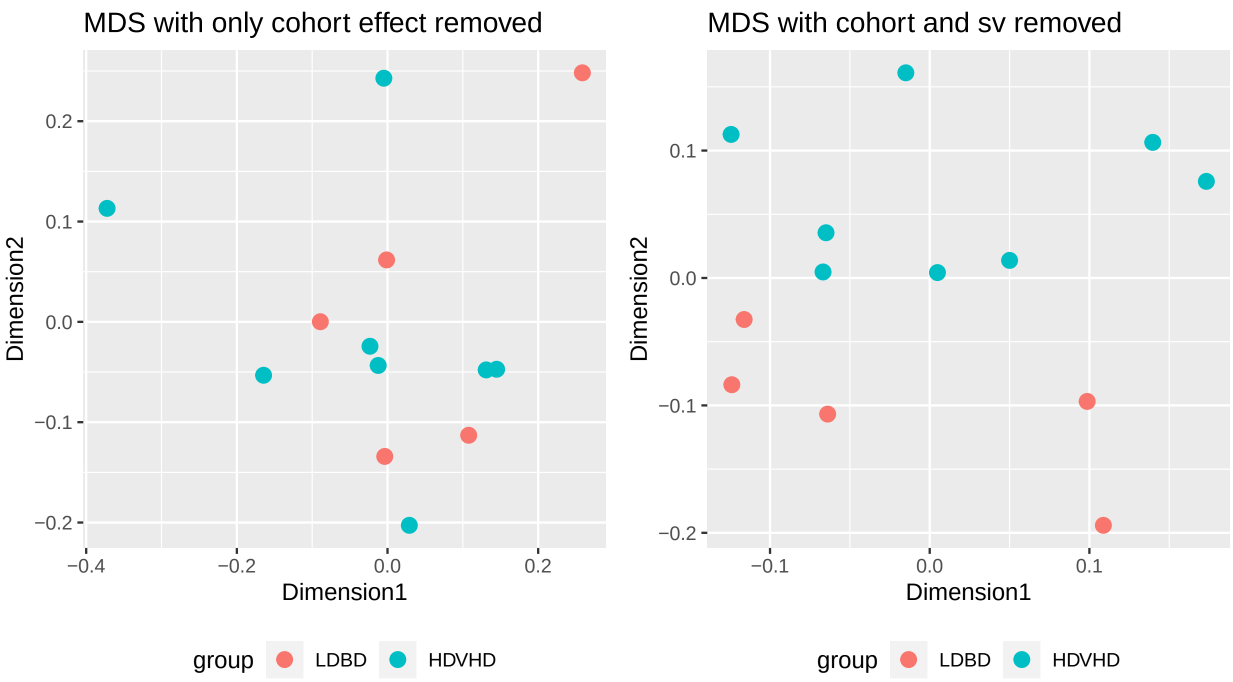

VHDvsHD_bio

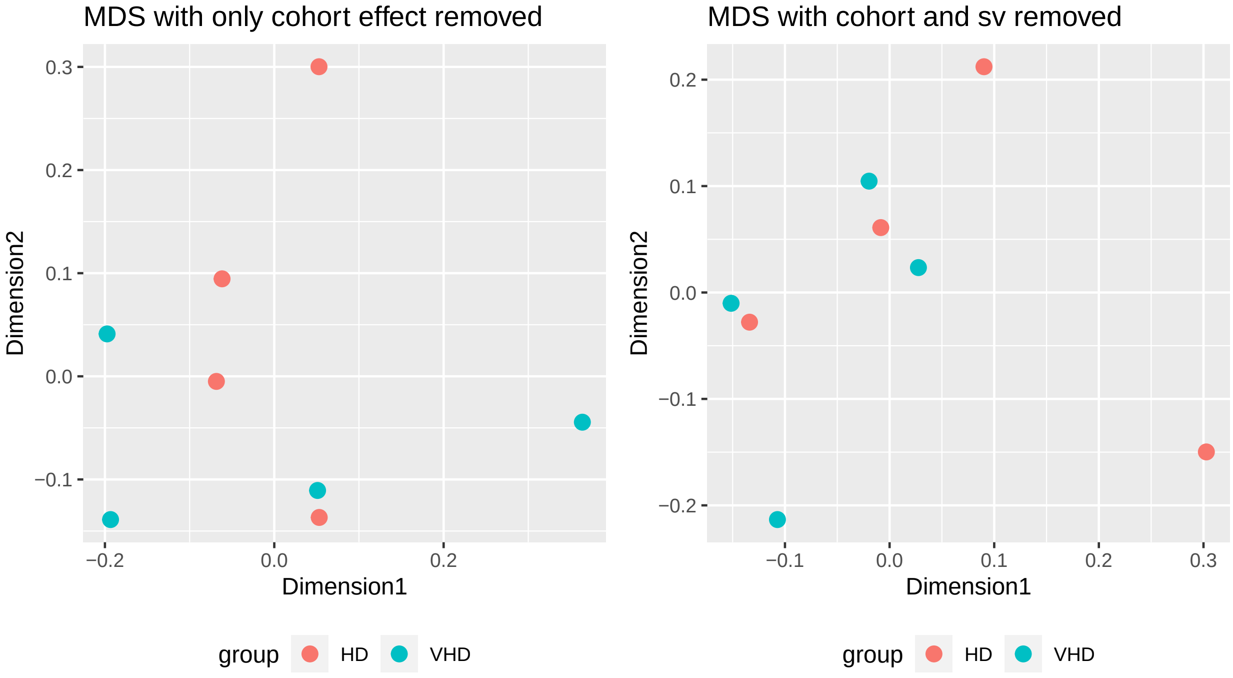

DRINKERvsCONTROL_nec

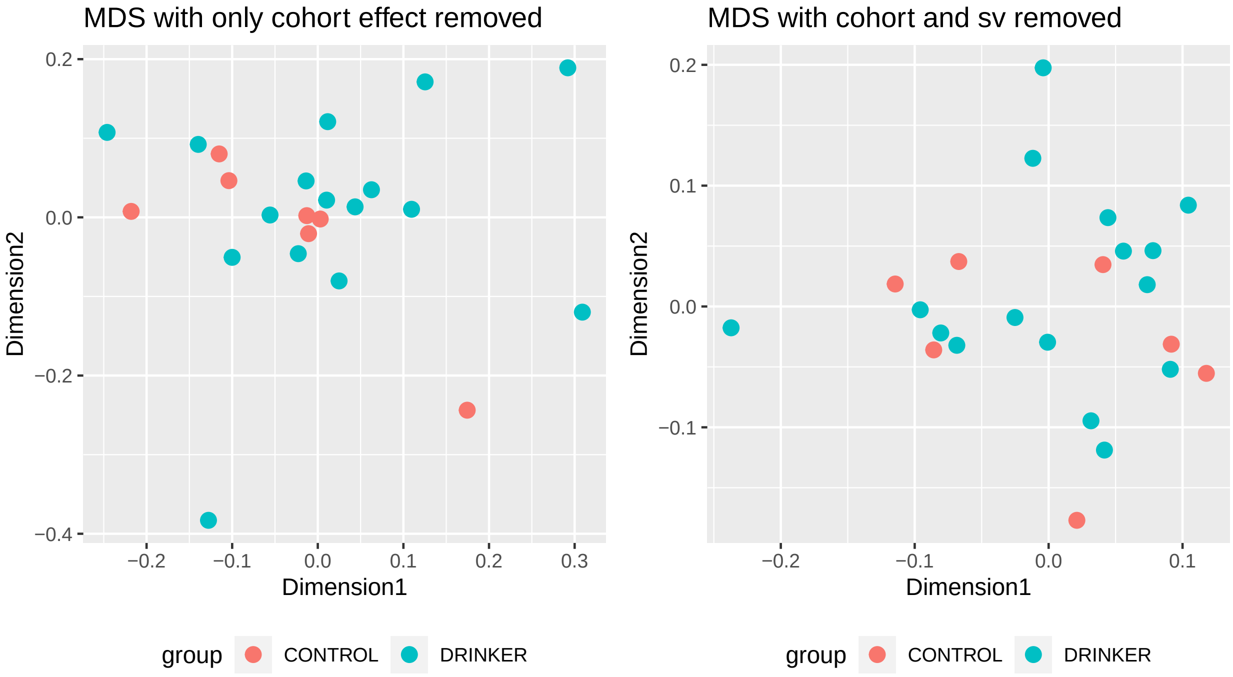

`VHDvsHD_nec`

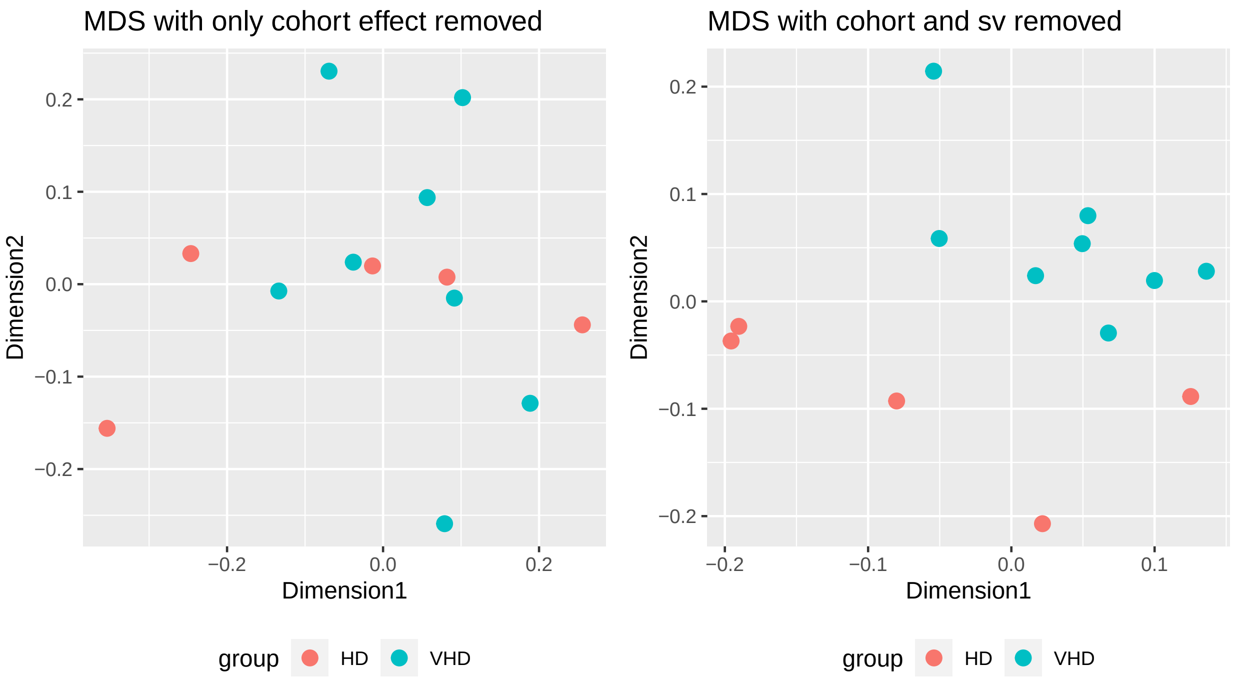

`control_nec.vs.bio`

Table 3: samples included in this analysis

SampleID	drGroup_open1_w_ctrl	drGroup_open3_w_ctrl	Timepoint
S10220_A12_L_nec_pre	control	control	nec
S10220_A12_R_bio_pre	control	control	bio
S10221_A12_L_bio_pre	control	control	bio
S10221_A12_R_nec_pre	control	control	nec
S10222_A12_L_nec_pre	control	control	nec
S10222_A12_R_bio_pre	control	control	bio
S10223_A12_L_bio_pre	control	control	bio
S10223_A12_R_nec_pre	control	control	nec
S10241_A46_biop_post_etoh	control	control	nec
S10241_A46_biop_pre_etoh	control	control	bio
S10245_A46_biop_post_etoh	control	control	nec
S10245_A46_biop_pre_etoh	control	control	bio
S10250_A46_biop_post_etoh	control	control	nec
S10250_A46_biop_pre_etoh	control	control	bio

`HD.to.VHD_nec.vs.bio`

Table 4: samples included in this analysis

SampleID	drGroup_open1_w_ctrl	drGroup_open3_w_ctrl	Timepoint
S10212_A12_L_bio_pre	HD	VHD	bio
S10212_A12_R_nec_pre	HD	VHD	nec
S10244_A46_biop_post_etoh	HD	VHD	nec
S10244_A46_biop_pre_etoh	HD	VHD	bio
S10247_A46_biop_post_etoh	HD	VHD	nec
S10247_A46_biop_pre_etoh	HD	VHD	bio
S10249_A46_biop_post_etoh	HD	VHD	nec
S10249_A46_biop_pre_etoh	HD	VHD	bio

`VHD.to.VHD_nec.vs.bio`

Table 5: samples included in this analysis

SampleID	drGroup_open1_w_ctrl	drGroup_open3_w_ctrl	Timepoint
S10215_A12_L_nec_pre	VHD	VHD	nec
S10215_A12_R_bio_pre	VHD	VHD	bio
S10242_A46_biop_post_etoh	VHD	VHD	nec
S10242_A46_biop_pre_etoh	VHD	VHD	bio
S10248_A46_biop_post_etoh	VHD	VHD	nec
S10248_A46_biop_pre_etoh	VHD	VHD	bio
S10252_A46_biop_post_etoh	VHD	VHD	nec
S10252_A46_biop_pre_etoh	VHD	VHD	bio

`BD.to.HD_nec.vs.bio`

Table 6: samples included in this analysis

SampleID	drGroup_open1_w_ctrl	drGroup_open3_w_ctrl	Timepoint
S10208_A12_R_nec_pre	BD	HD	nec
S10209_A12_R_nec_pre	BD	HD	nec
S10211_A12_L_bio_pre	BD	HD	bio
S10211_A12_R_nec_pre	BD	HD	nec
S10251_A46_biop_post_etoh	BD	HD	nec
S10251_A46_biop_pre_etoh	BD	HD	bio

`DRINKER_change_vs_CONTROL_change`

- All samples are included in this analysis

Summary of DE results and plots

Table 7: Number of DE genes in each comparison, FDR adjusted p-value <0.05

	Down	noChange	Up
DRINKERvsCONTROL_bio	0	13868	0
HDVHDvsLDBD_bio	0	13868	0
VHDvsHD_bio	0	13868	0
DRINKERvsCONTROL_nec	0	13868	0
VHDvsHD_nec	0	13868	0
control_nec.vs.bio	269	13144	455
HD.to.VHD_nec.vs.bio	14	13821	33
VHD.to.VHD_nec.vs.bio	1	13862	5
BD.to.HD_nec.vs.bio	0	13867	1
DRINKER_change_vs_CONTROL_change	0	13868	0

Table 8: Number of DE genes in each comparison, FDR adjusted p-value <0.2

	Down	noChange	Up
DRINKERvsCONTROL_bio	0	13868	0
HDVHDvsLDBD_bio	0	13868	0
VHDvsHD_bio	0	13868	0
DRINKERvsCONTROL_nec	0	13868	0
VHDvsHD_nec	0	13868	0
control_nec.vs.bio	1468	10757	1643
HD.to.VHD_nec.vs.bio	285	13195	388
VHD.to.VHD_nec.vs.bio	57	13719	92
BD.to.HD_nec.vs.bio	367	13277	224
DRINKER_change_vs_CONTROL_change	0	13868	0

Table 9: Number of DE genes in each comparison, nominal p-value <0.05

	Down	noChange	Up
DRINKERvsCONTROL_bio	448	12713	707
HDVHDvsLDBD_bio	306	13360	202
VHDvsHD_bio	170	13416	282
DRINKERvsCONTROL_nec	443	12905	520
VHDvsHD_nec	194	13514	160
control_nec.vs.bio	1537	10603	1728
HD.to.VHD_nec.vs.bio	1139	11575	1154
VHD.to.VHD_nec.vs.bio	803	12330	735
BD.to.HD_nec.vs.bio	1088	11959	821
DRINKER_change_vs_CONTROL_change	160	13423	285

log2FC, pearson's correlation

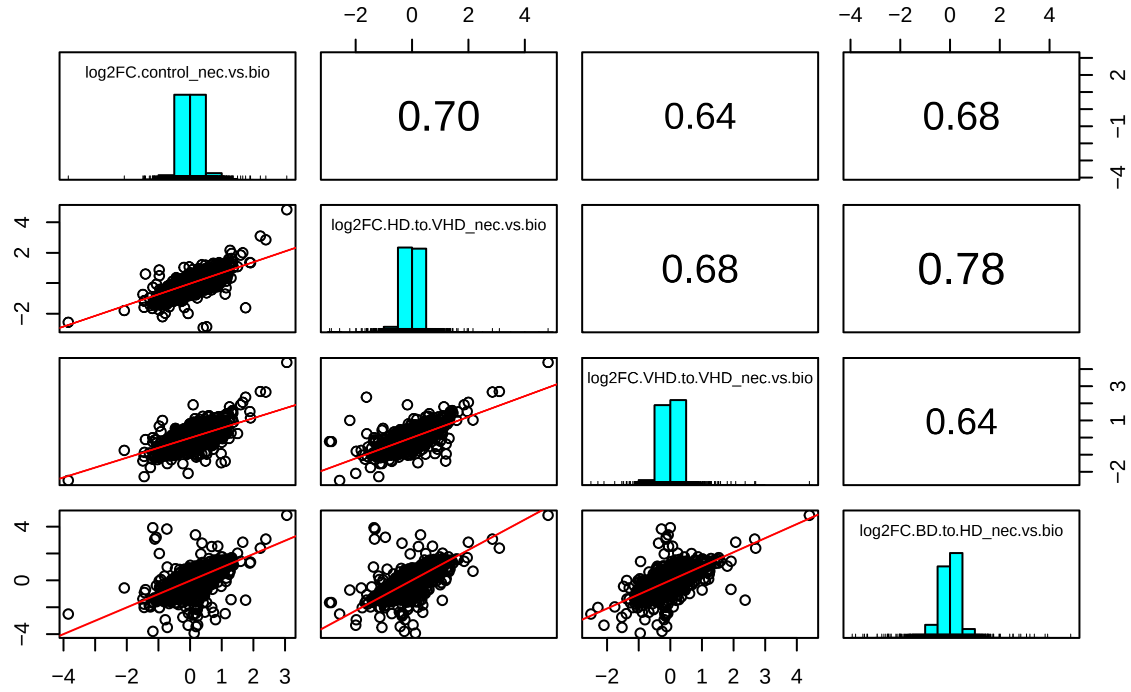

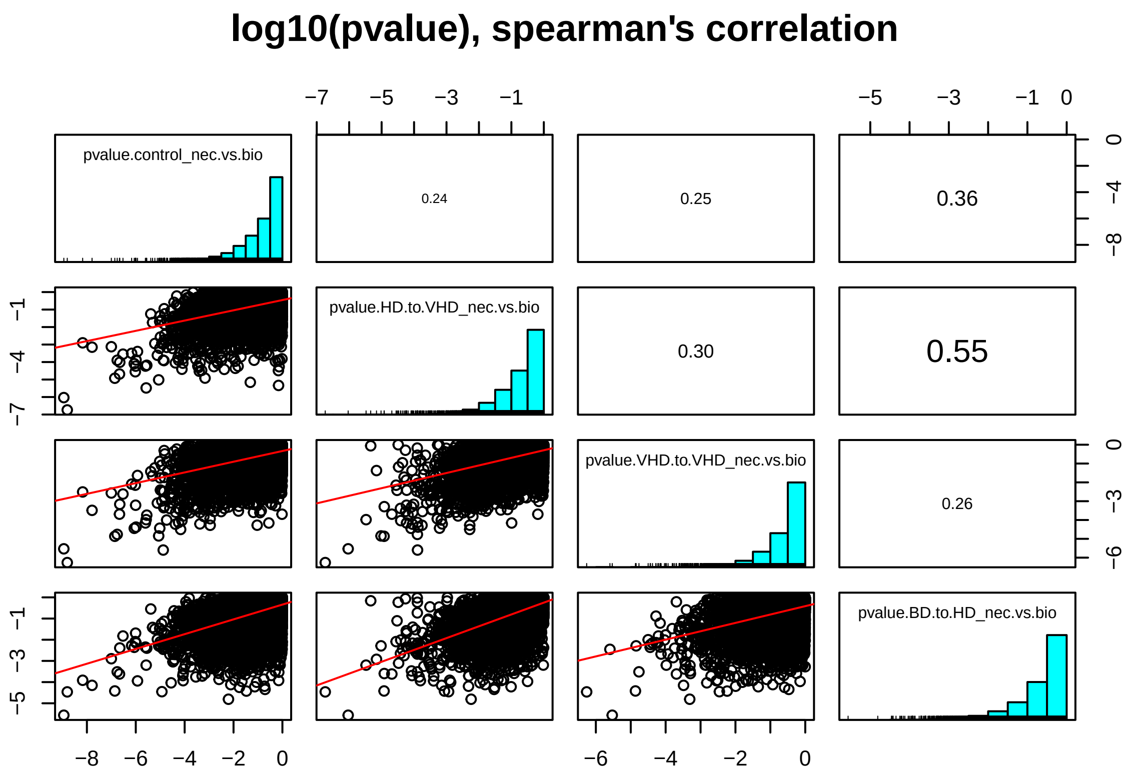

- As expected, all `nec.vs.bio` comparisons yielded similar expression profile, which suggests that the majority of genes that are changing in expression in drinker subgroups are probably not attributable to drinking. **The necropsy vs. biopsy comparison in controls should always be considered as reference when interpreting DE genes or pathways in drinker subgroups.**
- Using arbitrary DE thresholds, the number of DE genes is largest in controls. It is probably because control group has the largest sample size.
- Meaningful Venn diagrams are saved to `venn.pdf`, please let us know if additional venn diagrams are desired.
- volcano plots for all comparisons save in `volcano_plots.pdf`
- Meaningful heatmaps are saved to `heatmap_biopsy.pdf` and `heatmap_necropsy.pdf`, please let us know if additional heatmaps are desired.

Heat map of log2(normalized counts per million) of all genes in biopsy samples grouped by drinking category at open access 1

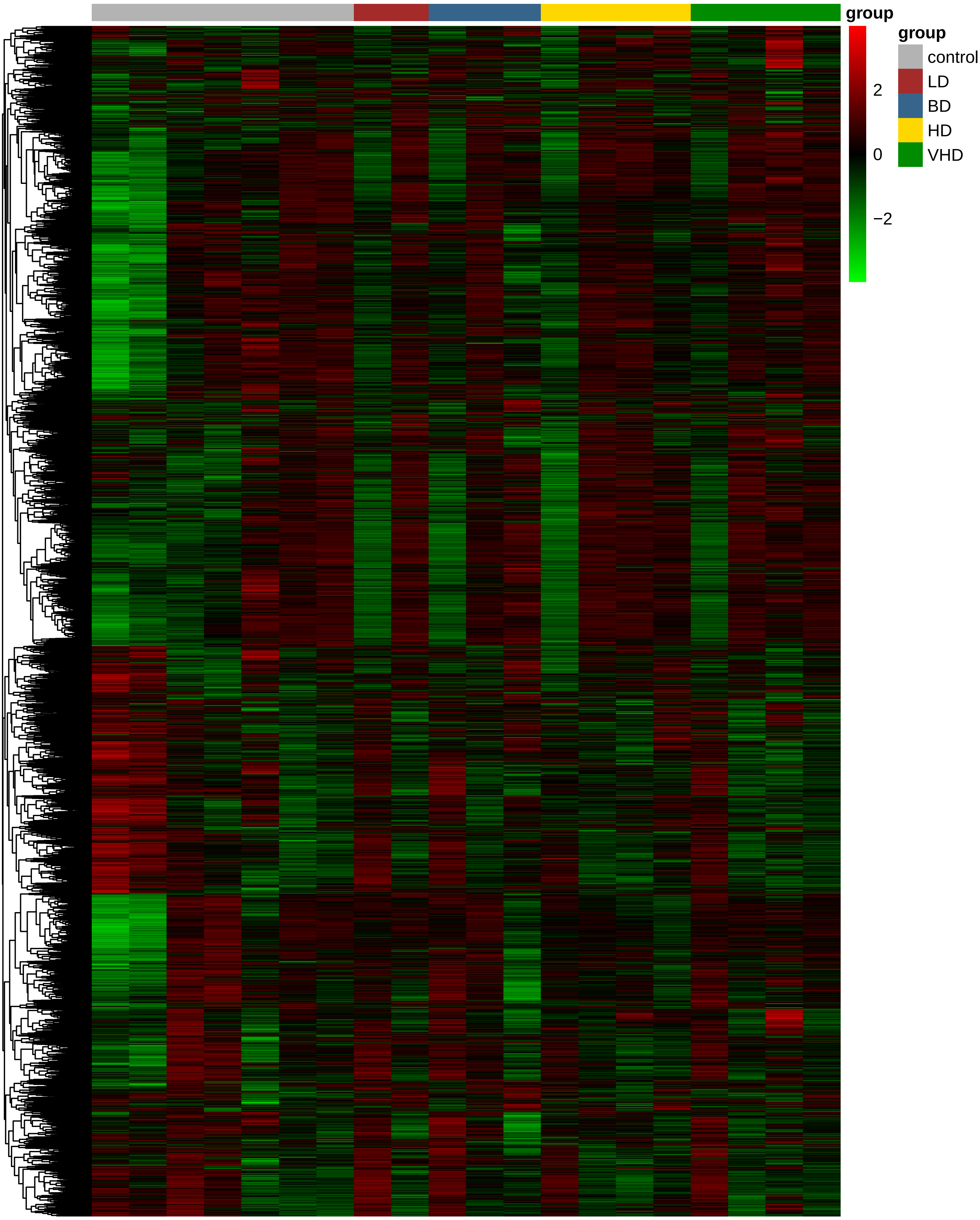

Heat map of log2(normalized counts per million) of all genes in necropsy samples

grouped by drinking category at open access 3

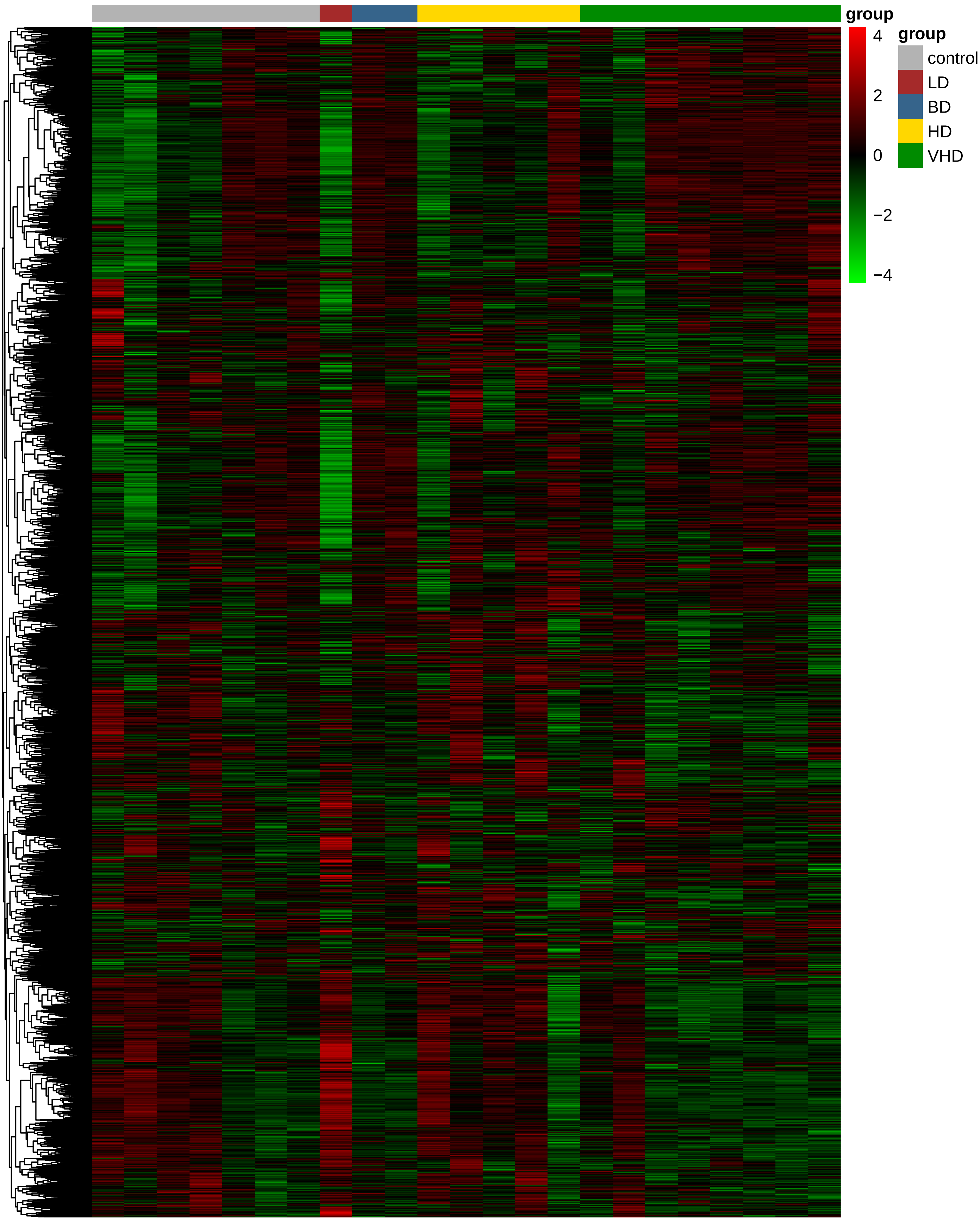

number of DE genes, FDR−adjusted pvalue < 0.2

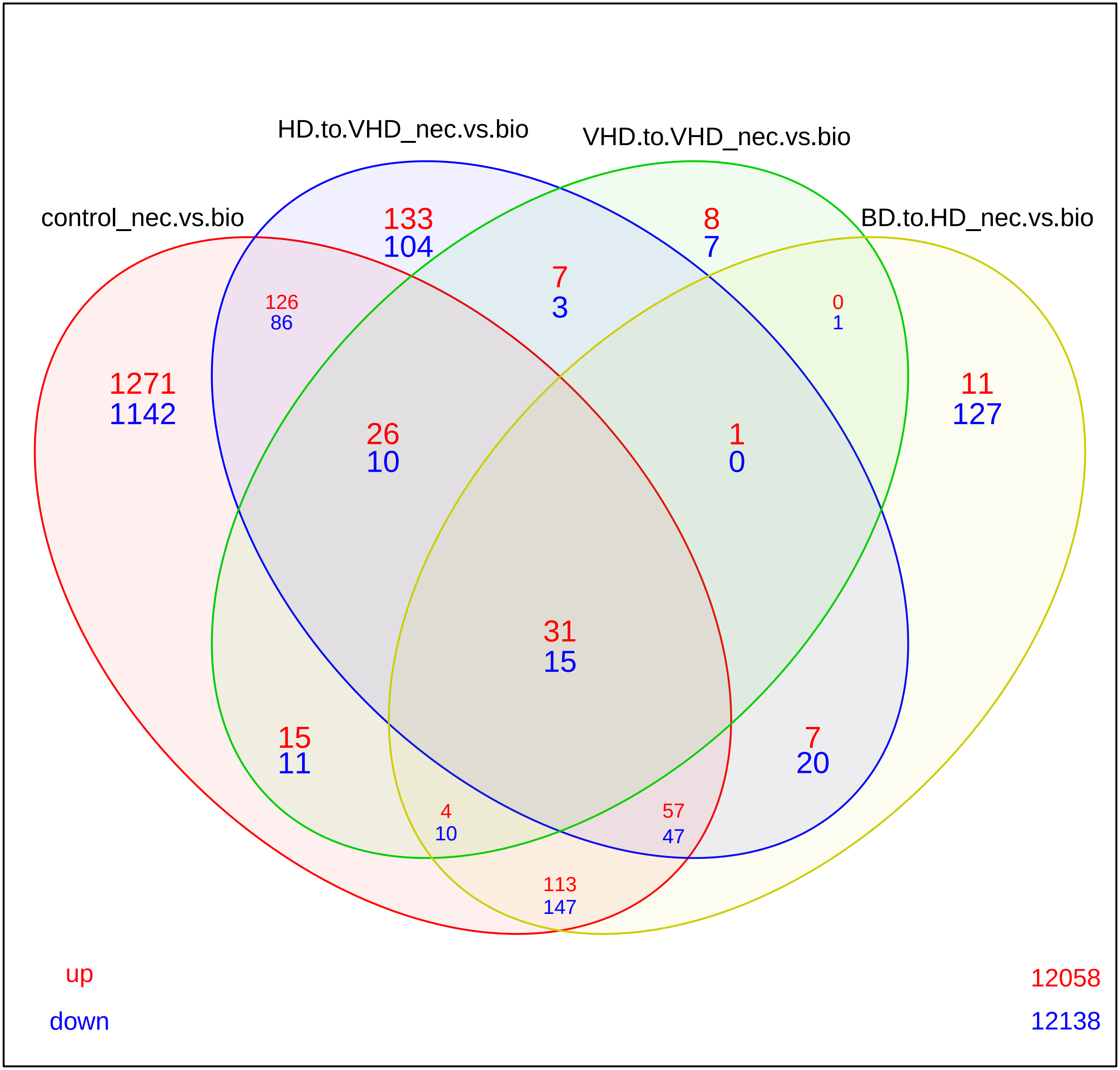

number of DE genes, nominal p−value < 0.05

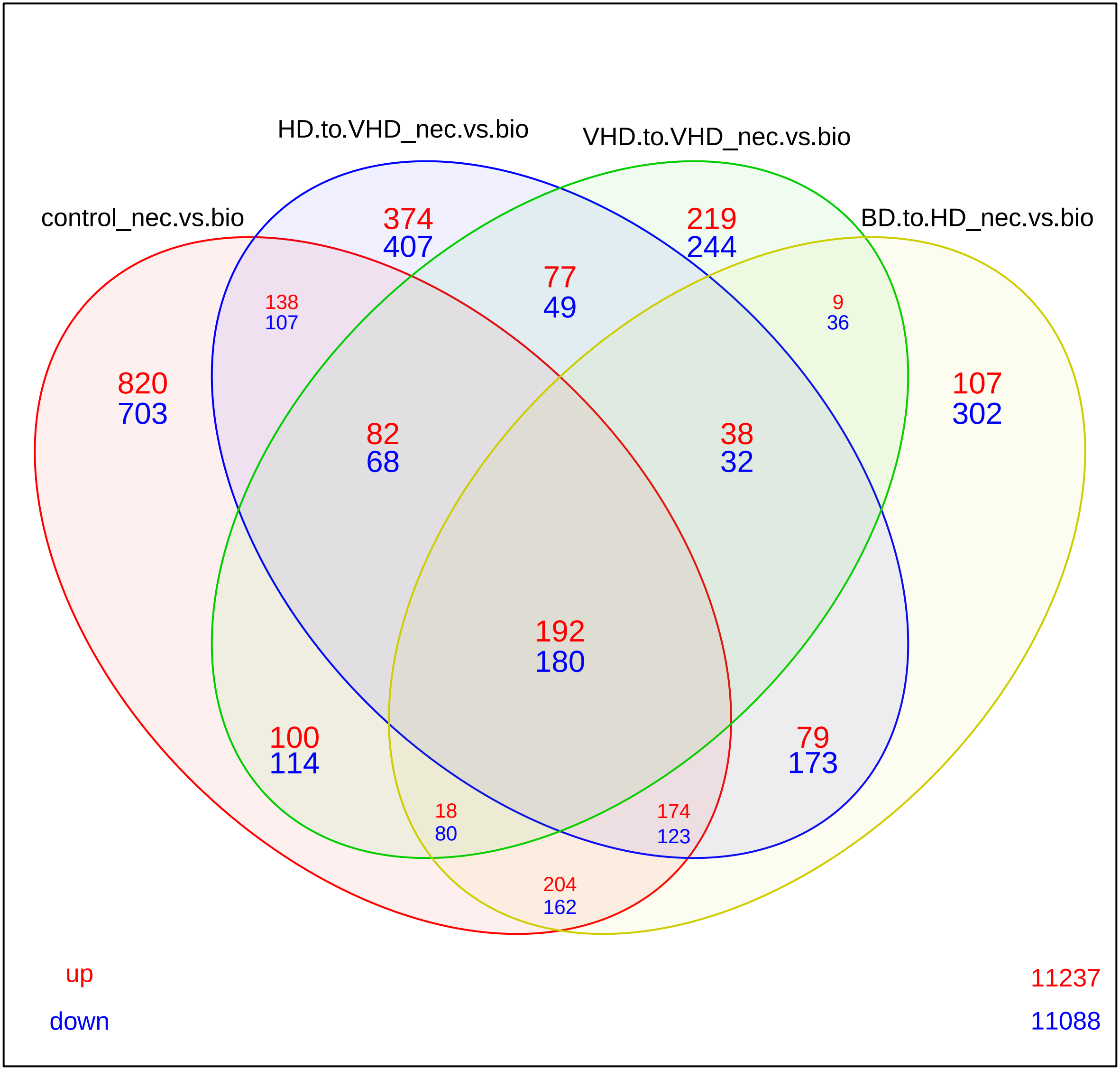

number of DE genes, FDR−adjusted pvalue < 0.2

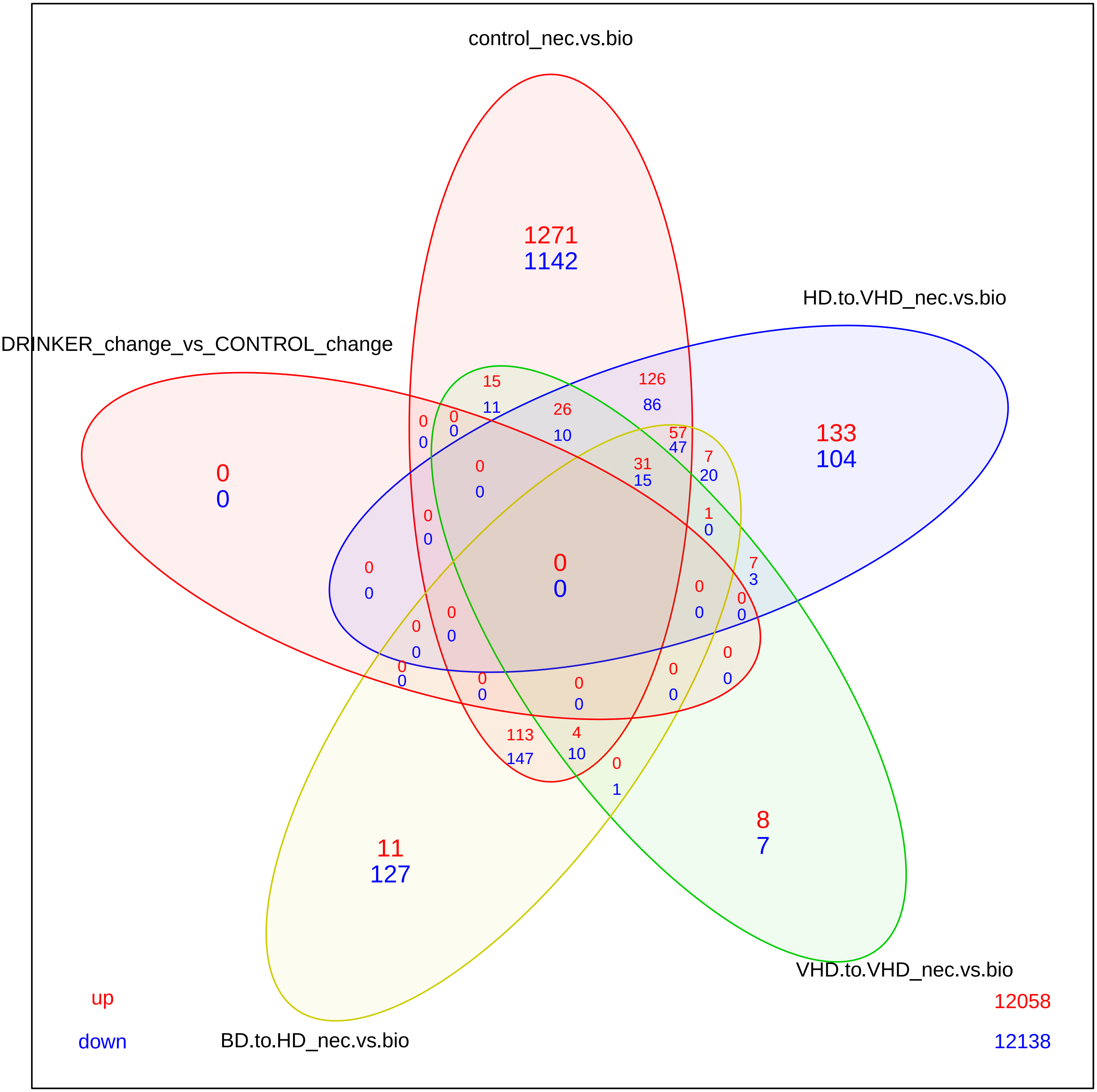

number of DE genes, FDR−adjusted pvalue < 0.05

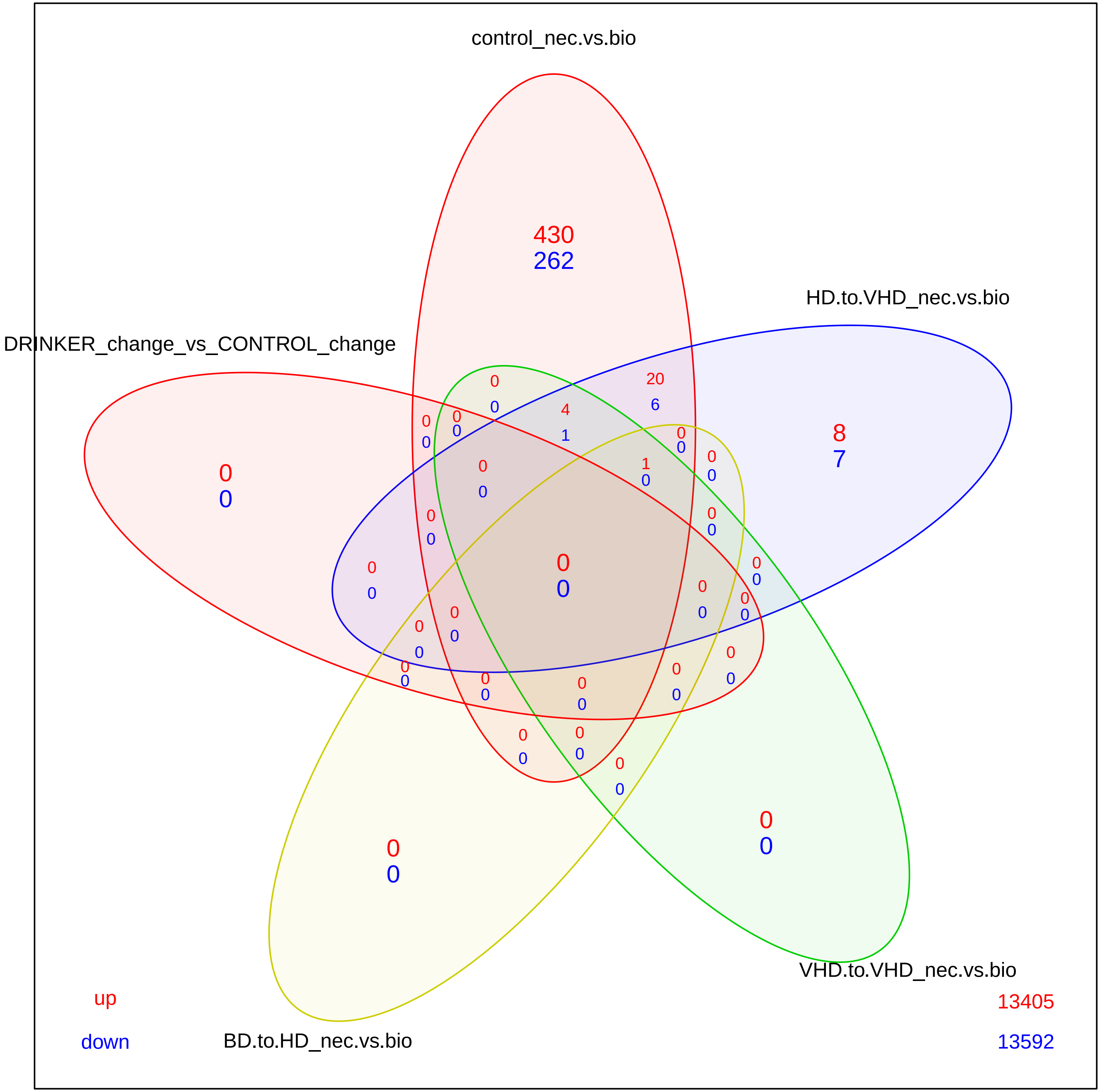

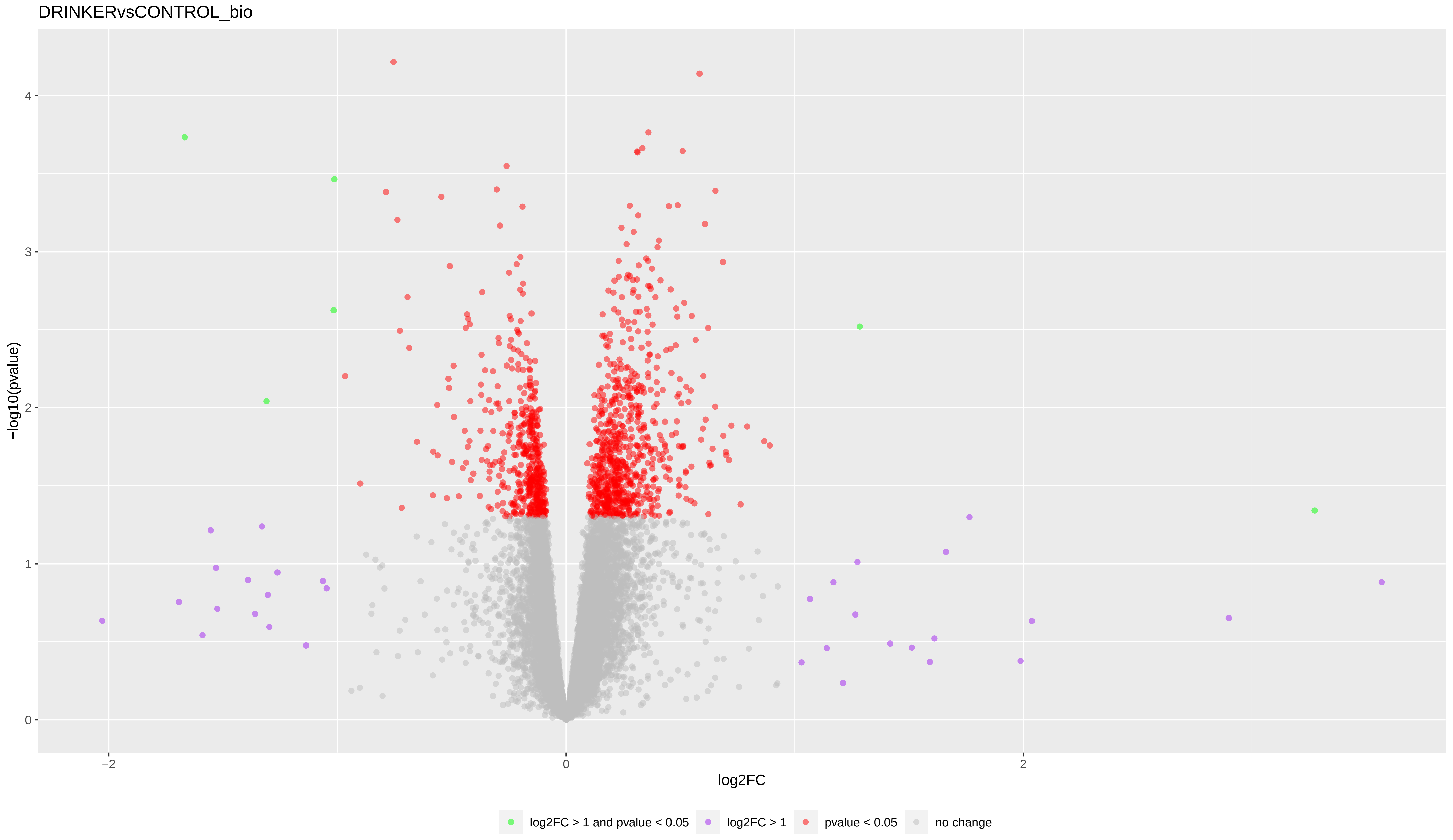
DRINKERvsCONTROL_bio
4
3
2
1
0
−log10(pvalue)
−2
0
2
log2FC
log2FC > 1 and pvalue < 0.05
log2FC > 1
pvalue < 0.05
no change

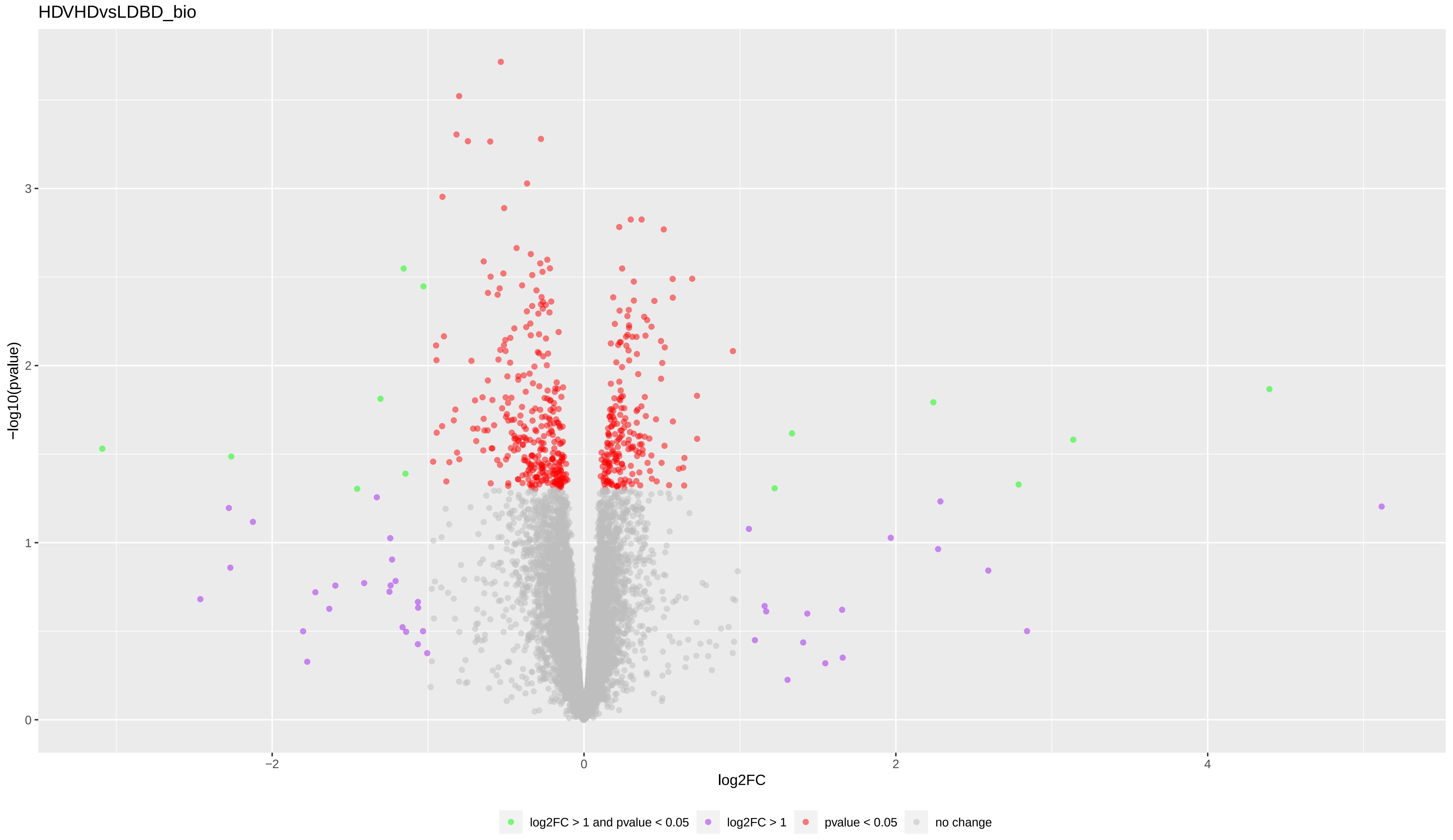
HDVHDvsLDBD_bio
−log10(pvalue)
3
2
1
0
−2
0
2
4
log2FC
log2FC > 1 and pvalue < 0.05
log2FC > 1
pvalue < 0.05
no change

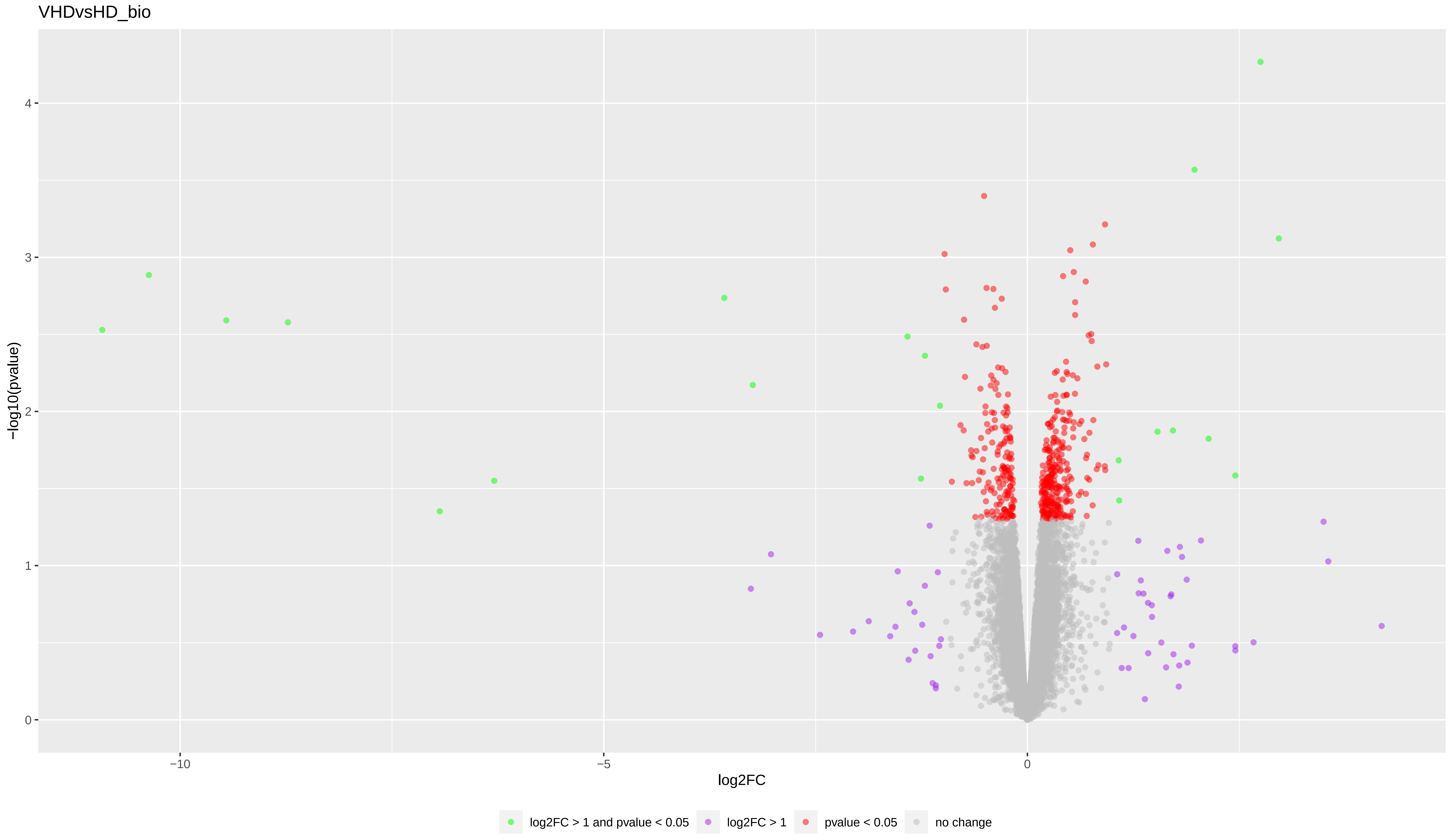
VHDvsHD_bio
−log10(pvalue)
0
1
2
3
4
−10
−5
0
log2FC
log2FC > 1 and pvalue < 0.05
log2FC > 1
pvalue < 0.05
no change

DRINKERvsCONTROL_nec

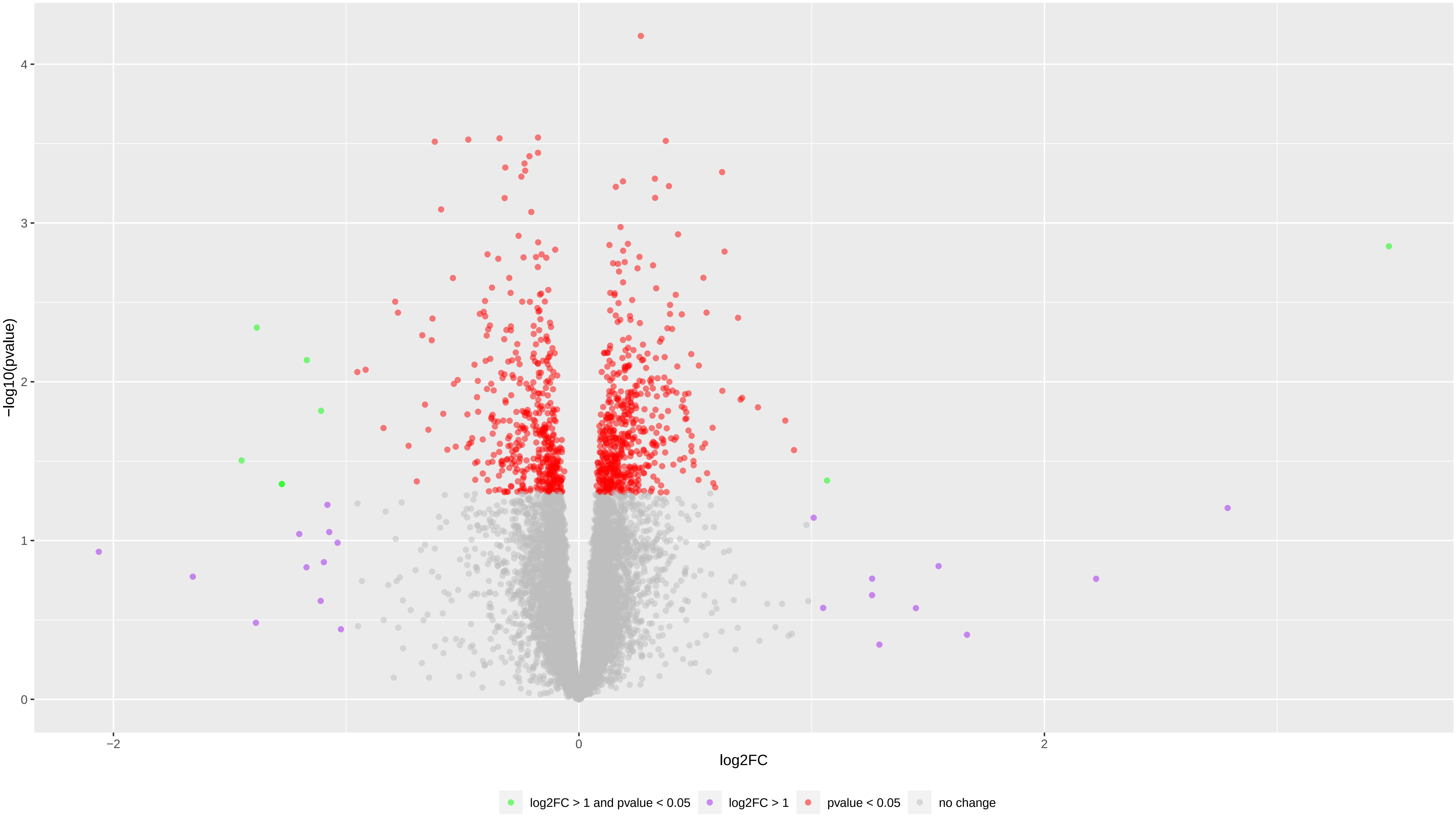

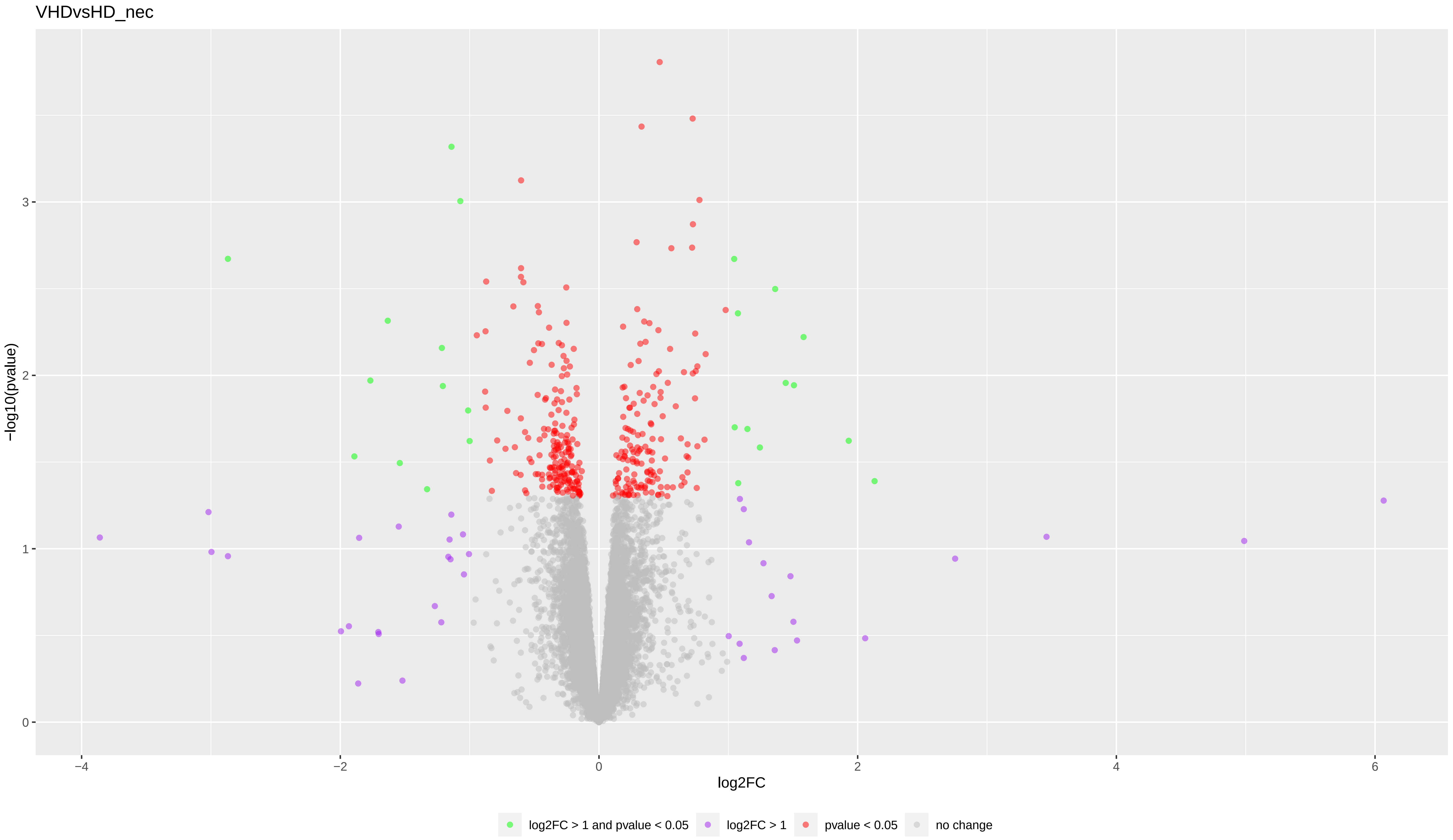
VHDvsHD_nec
−log10(pvalue)
0
1
2
3
−4
−2
0
2
4
6
log2FC
log2FC > 1 and pvalue < 0.05
log2FC > 1
pvalue < 0.05
no change

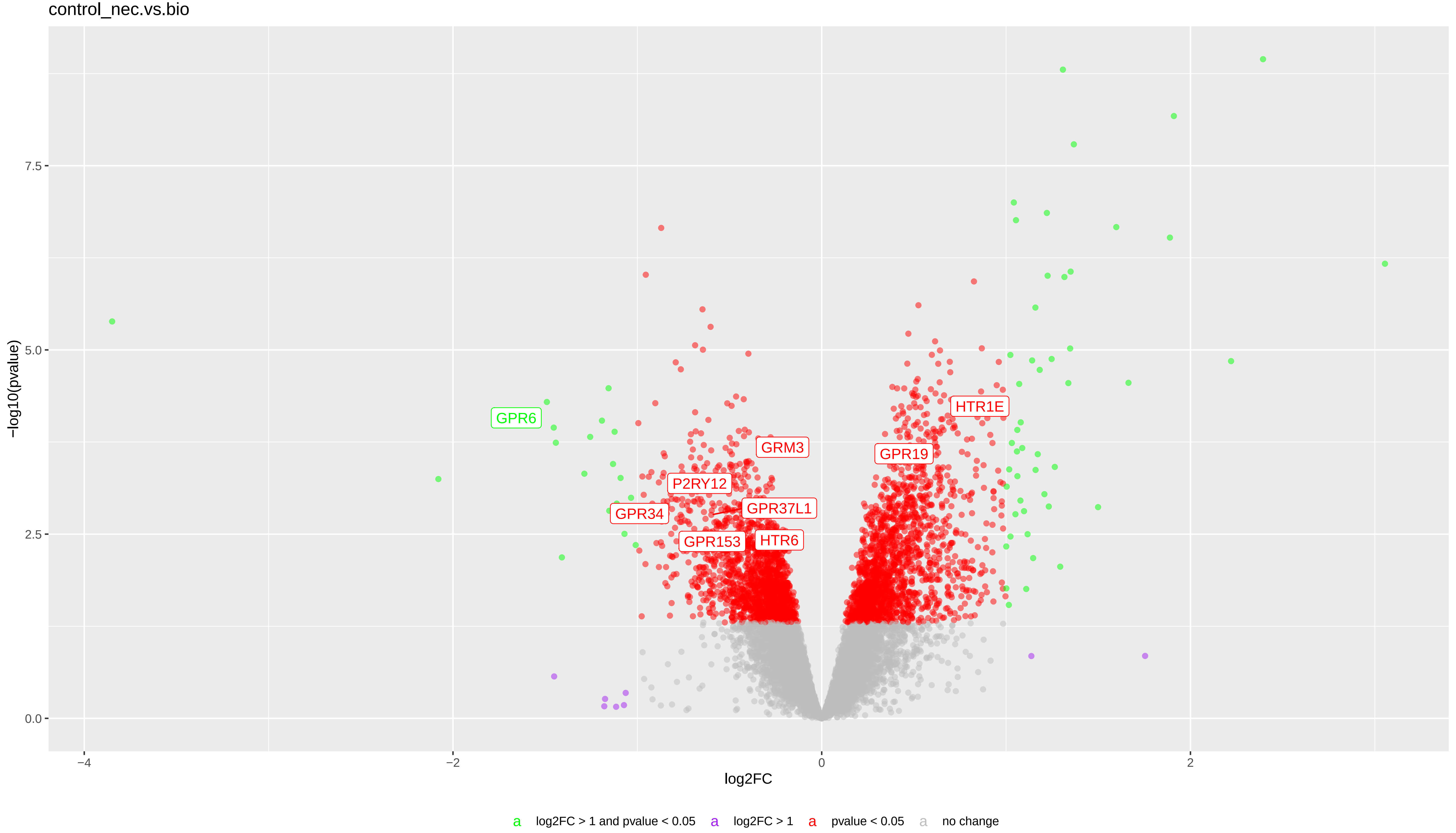
control_nec.vs.bio
7.5
5.0
2.5
0.0
−log10(pvalue)
−4
−2
0
2
log2FC
GPR6
GRM3
GPR19
HTR1E
P2RY12
GPR34
GPR37L1
GPR153
HTR6
a log2FC > 1 and pvalue < 0.05
a log2FC > 1
a pvalue < 0.05
a no change

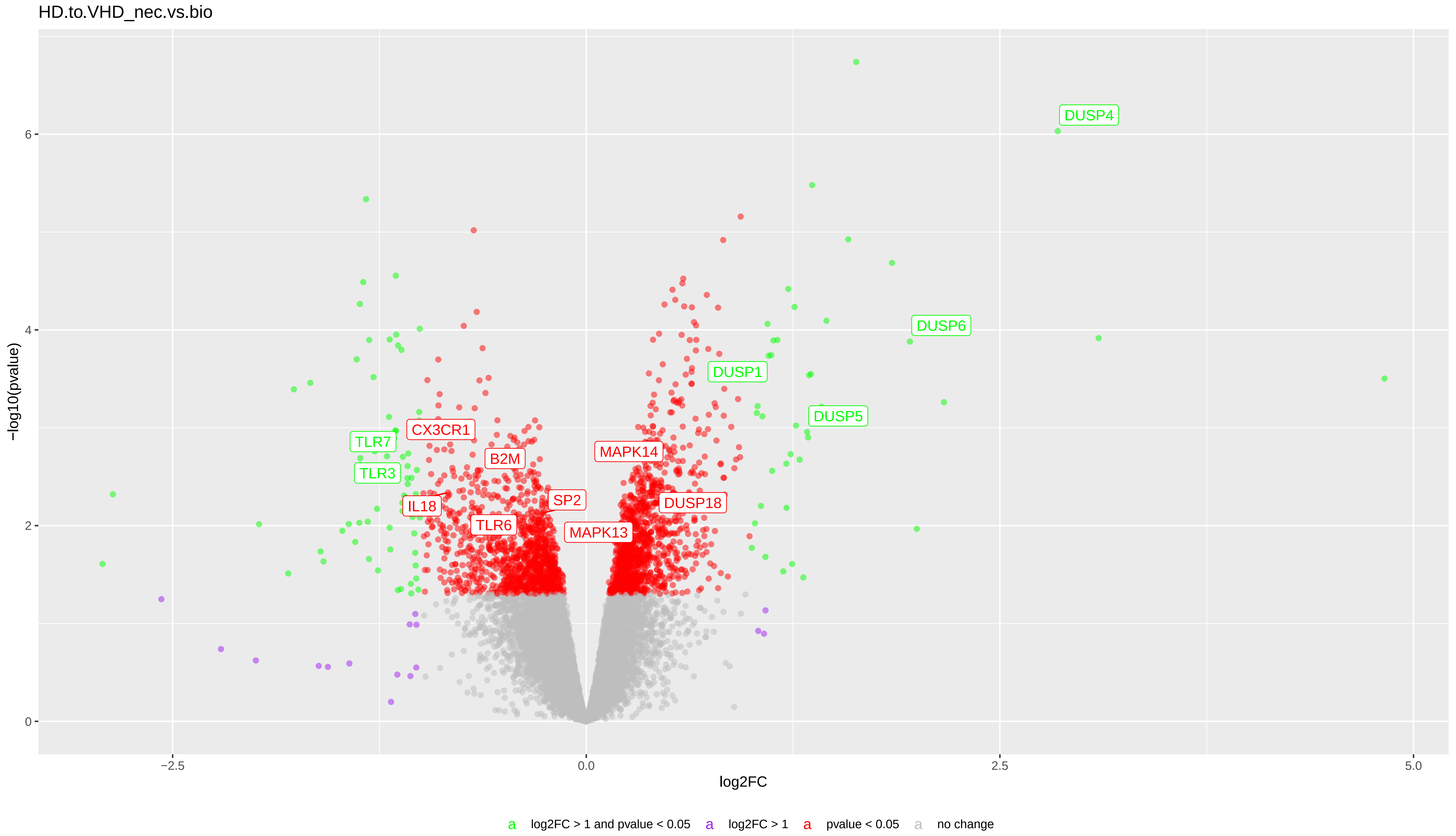
HD.to.VHD_nec.vs.bio
DUSP4
DUSP6
DUSP1
DUSP5
TLR7
TLR3
CX3CR1
B2M
IL18
TLR6
SP2
MAPK14
MAPK13
DUSP18
-log10(pvalue)
log2FC
0
2
4
6
−2.5
0.0
2.5
5.0
a log2FC > 1 and pvalue < 0.05
a log2FC > 1
a pvalue < 0.05
a no change

VHD.to.VHD_nec.vs.bio

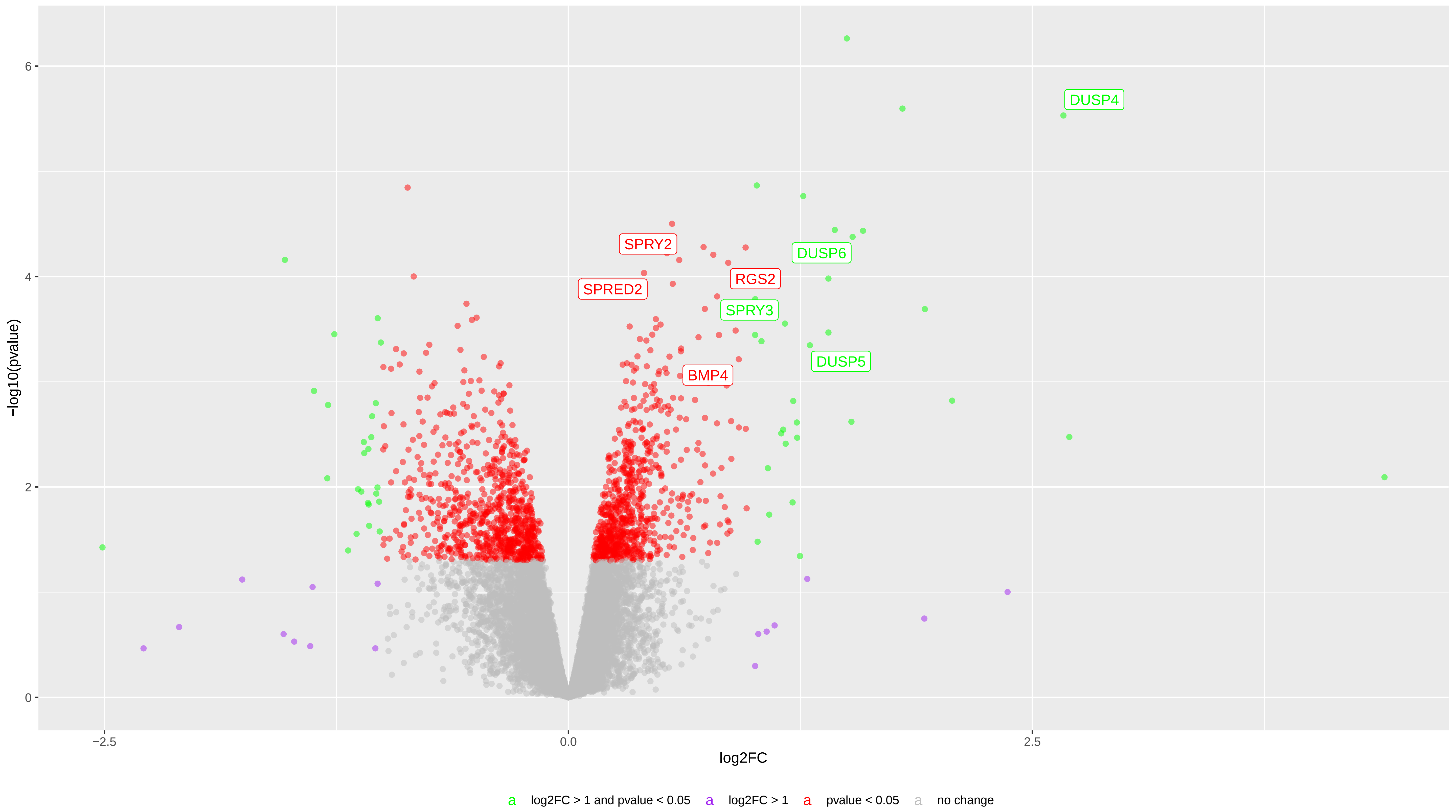

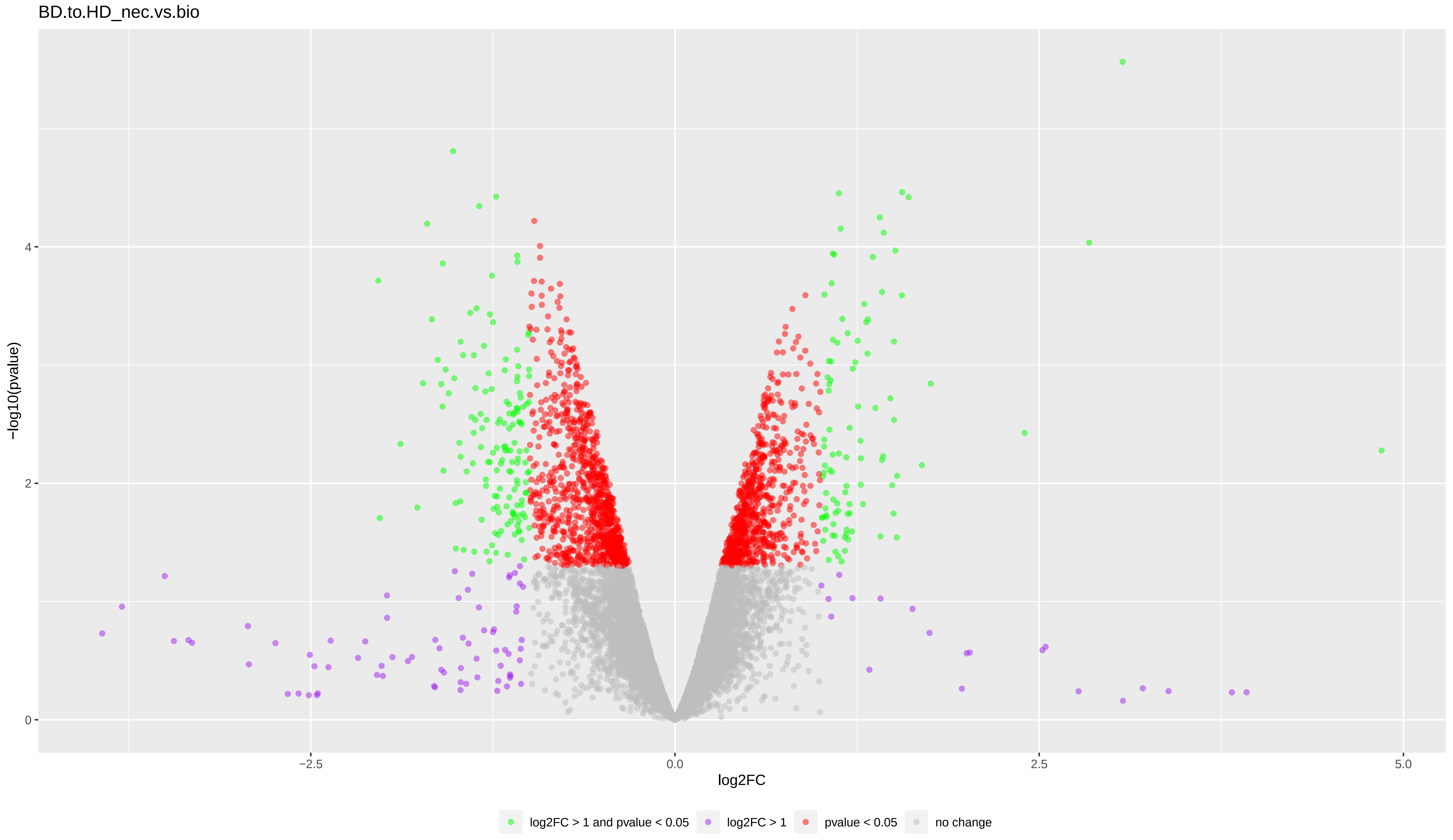
BD.to.HD_nec.vs.bio
−log10(pvalue)
4
2
0
−2.5
0.0
2.5
5.0
log2FC
log2FC > 1 and pvalue < 0.05
log2FC > 1
pvalue < 0.05
no change

DRINKER_change_vs_CONTROL_change

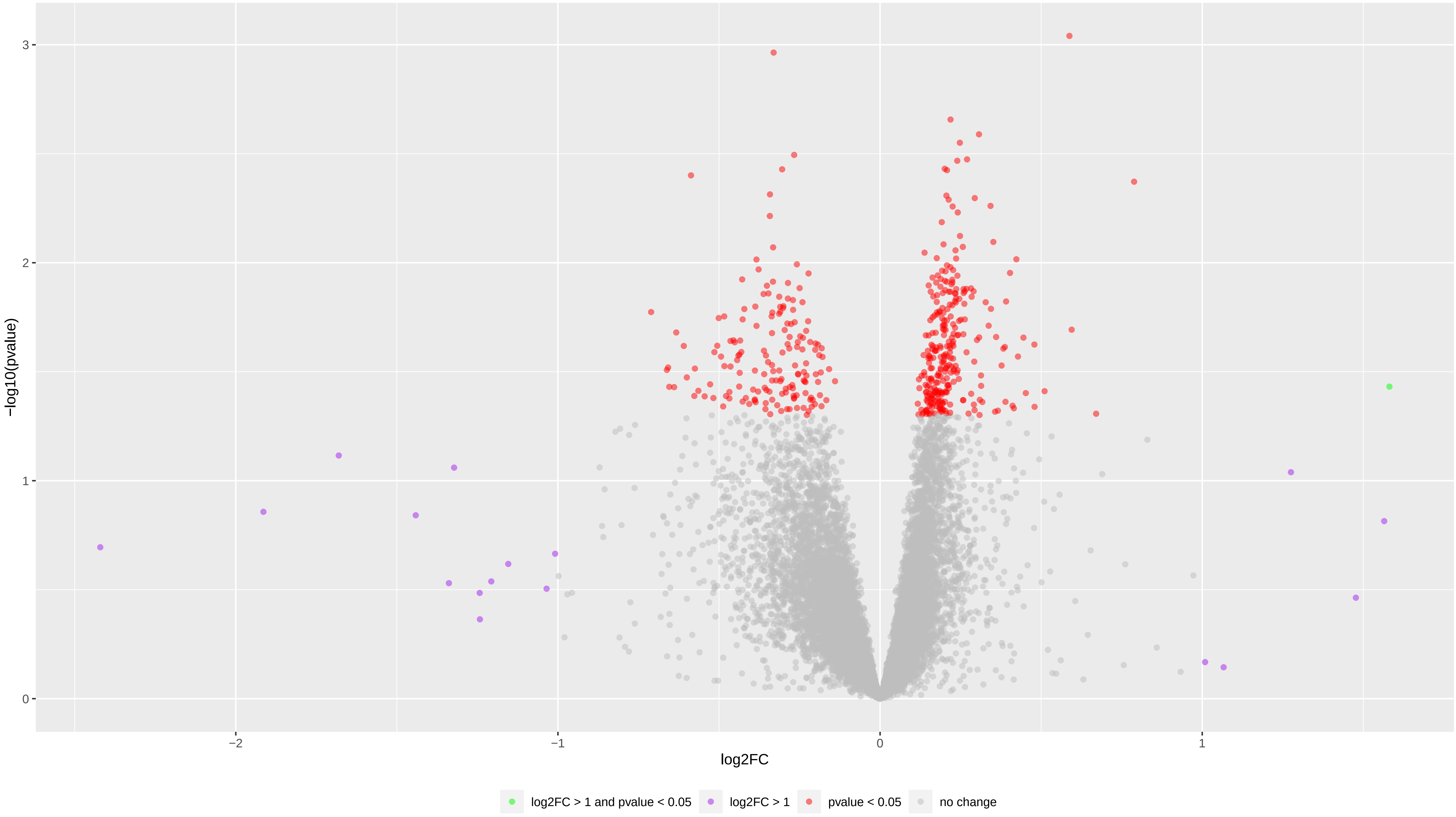

Supplement: Supplementary file 3 [file DataSheet1.PDF]
